# The adult shell matrix protein repertoire of the marine snail *Crepidula* is dominated by conserved genes that are also expressed in larvae

**DOI:** 10.1186/s12862-024-02237-y

**Published:** 2024-09-14

**Authors:** Rebecca N. Lopez-Anido, Grant O. Batzel, Gabriela Ramirez, Yiqun Wang, Stephanie Neal, Maryna P. Lesoway, Jessica A. Goodheart, Deirdre C. Lyons

**Affiliations:** 1 Scripps Institution of Oceanography, U.C. San Diego, La Jolla, CA USA; 2 Division of Invertebrate Zoology, American Museum of Natural History, New York, NY USA

**Keywords:** Crepidula, Shell development, Biomineralization, Shell matrix proteins

## Abstract

Mollusca is a morphologically diverse phylum, exhibiting an immense variety of calcium carbonate structures. Proteomic studies of adult shells often report high levels of rapidly-evolving, ‘novel’ shell matrix proteins (SMPs), which are hypothesized to drive shell diversification. However, relatively little is known about the phylogenetic distribution of SMPs, or about the function of individual SMPs in shell construction. To understand how SMPs contribute to shell diversification a thorough characterization of SMPs is required. Here, we build tools and a foundational understanding of SMPs in the marine gastropod species *Crepidula fornicata* and *Crepidula atrasolea* because they are genetically-enabled mollusc model organisms. First, we established a staging system of shell development in *C. atrasolea* for the first time. Next, we leveraged previous findings in *C. fornicata* combined with phylogenomic analyses of 95 metazoan species to determine the evolutionary lineage of its adult SMP repertoire. We found that 55% of *C. fornicata’s* SMPs belong to molluscan orthogroups, with 27% restricted to Gastropoda, and only 5% restricted at the species level. The low percentage of species-restricted SMPs underscores the importance of broad-taxon sampling and orthology inference approaches when determining homology of SMPs. From our transcriptome analysis, we found that the majority of *C. fornicata* SMPs that were found conserved in *C. atrasolea* were expressed in both larval and adult stages. We then selected a subset of SMPs of varying evolutionary ages for spatial-temporal analysis using in situ hybridization chain reaction (HCR) during larval shell development in *C. atrasolea*. Out of the 18 SMPs analyzed, 12 were detected in the larval shell field. These results suggest overlapping larval vs. adult SMP repertoires. Using multiplexed HCR, we observed five SMP expression patterns and three distinct cell populations within the shell field. These patterns support the idea that modular expression of SMPs could facilitate divergence of shell morphological characteristics. Collectively, these data establish an evolutionary and developmental framework in *Crepidula* that enables future comparisons of molluscan biomineralization to reveal mechanisms of shell diversification.

**Supplementary Information:**

The online version contains supplementary material available at 10.1186/s12862-024-02237-y.

## Background

**Fig. 1:**
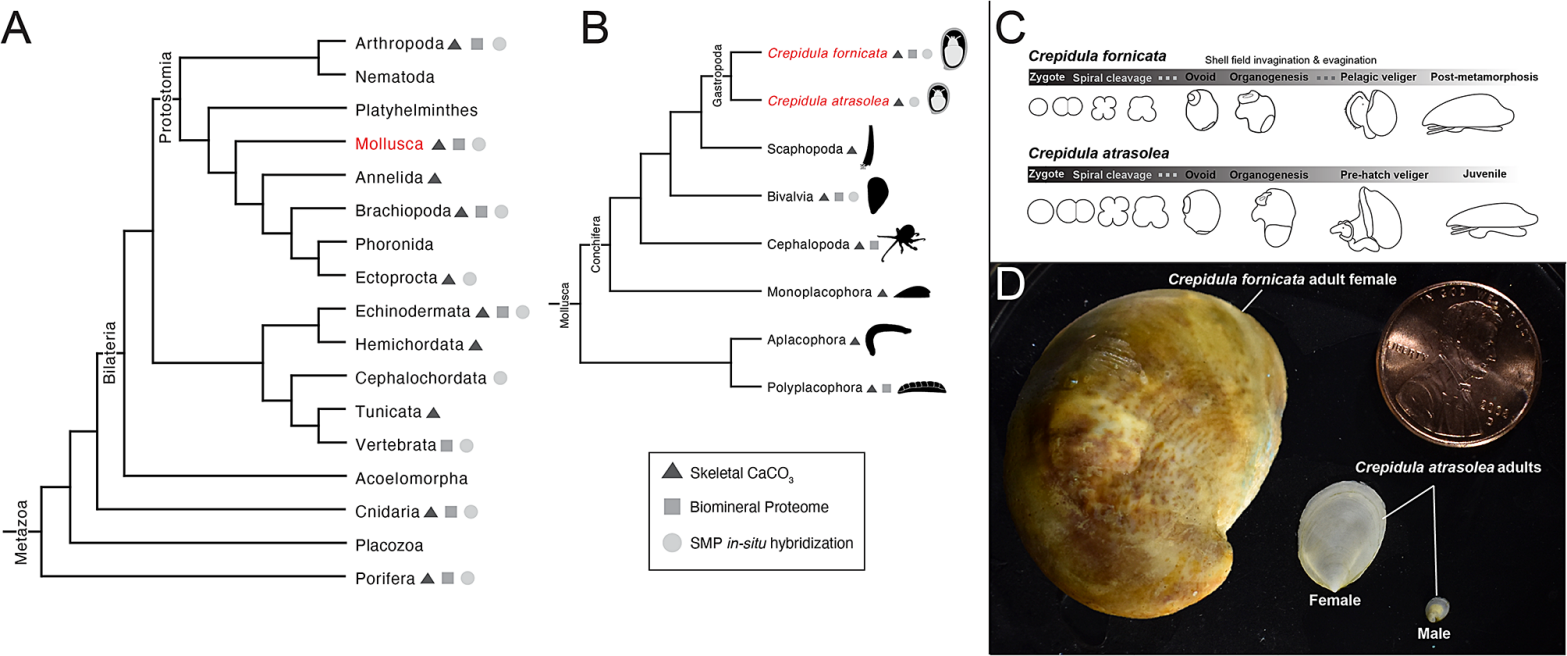
*Crepidula fornicata* and *Crepidula atrasolea* are complementary model systems for biomineralization. Phylogeny of calcium carbonate biomineralization throughout the Metazoa (**A**; Gilbert et al. 2022) and the Mollusca (**B**; Kocot et al. 2020). Triangles indicate taxa that produce calcium carbonate skeletons; squares indicate taxa that have a published biomineral proteome as of 2023; circles indicate taxa that have conducted in situ hybridization for shell matrix proteins (SMPs). Comparison of indirect larval development in *C. fornicata* vs. direct development in *C. atrasolea* (**C**). Adult shells of the two species (**D**). Organism silhouettes are from PhyloPic (www.phylopic.org) with the exception of *Crepidula* and *Polyplacophora*

Calcium carbonate biomineralization is a significant innovation in the evolution of divergent body forms, and is thought to have evolved independently at least 20 times in metazoans (Fig. 1A) [1–3]. Within the phylum Mollusca alone it is hypothesized that calcium carbonate biomineralization evolved multiple times, contributing to a rich diversity of shell shape, size, color, microstructure, and mineral content (Fig. 1B) [4–10]. During molluscan shell biomineralization, specialized epithelial cells in the mantle tissue secrete organic materials into an enclosed extrapallial space, including polysaccharides, proteoglycans, and shell matrix proteins (SMPs) [11, 12]. Secreted SMPs self-assemble to create an extracellular matrix that facilitates the precipitation of calcium carbonate, and SMPs become occluded in the shell [13–15]. Shell proteomic comparative studies have identified only a few SMPs with homologs across molluscan species [16], suggesting that SMP repertoires include many rapidly evolving, lineage-specific proteins [5, 17]. The presence of lineage-specific SMPs has led to the hypothesis that at the protein structure level, SMP diversity may have driven shell diversification and contributed to the acquisition of novel shell morphologies [6, 12, 18]. One approach to test this hypothesis is to (1) identify a species’ SMP repertoire through proteomics, (2) determine the evolutionary age of its SMPs, (3) determine the spatiotemporal expression patterns of the SMPs across life history stages, and (4) functionally perturb conserved and lineage-restricted SMP gene products to assess the morphological consequences of losing specific SMPs. All four lines of analysis should be feasible in the same organism. Two such species are the marine slipper snails *Crepidula fornicata* and *Crepidula atrasolea* [19–22]. In the present study we determine the evolutionary age of all known *C. fornicata* SMPs and examine the expression patterns for a subset of lineage-specific SMPs during larval shell development. These results set the stage for future work to determine how SMPs may contribute to novel shell morphology and evolution.

While many studies examine SMPs that are occluded in adult shells, it is also important to study SMPs that are occluded in the larval shell. In gastropods, larval shell formation begins after gastrulation when the posterior dorsal epithelium thickens and becomes known as the shell field [23]. In the literature, “shell field” and “larval mantle” both refer to the larval tissues responsible for shell formation [23–26]. The shell field (which will eventually mature into the adult mantle tissue) secretes SMPs throughout larval development when shell morphology is first established [23]. Larval shells and adult shells can differ in microstructure, mineral content, and SMP composition [27–30]. Most studies identify SMPs use adult shells as the source material and relatively few studies focus on comparing SMP repertoires at different life history stages. While several studies highlight differences in the SMP content of larval vs. adult shells (e.g. in bivalves [29–31], similarities have also been identified. For example, we recently showed that several of the most highly-expressed adult *C. fornicata* SMPs are also expressed in the larval shell field [19]. Although functional perturbation is required to conclusively determine gene function, the expression of adult SMPs in the larval shell field is highly suggestive of a role in shell biomineralization. By examining SMPs during larval shell development, when shell morphology is first established, we can study the role of SMPs in the acquisition of new shell morphologies and shell diversity over developmental time. The deployment of SMPs during development could change over evolutionary time scales, contributing to shell diversity.

Shell diversification may also rely on the division of the mantle tissue into discrete regions of SMP expression, with each region responsible for producing specific shell characteristics [24, 32]. Distinct regions of SMP expression within the mantle have been observed in multiple molluscan species [24, 33, 34]. For example, in adult bivalves, SMPs involved in nacreous-layer formation are found in the dorsal region of the mantle epithelium [34, 35], whereas SMPs implicated in prismatic-layer biomineralization are localized to the ventral portion of the mantle epithelium [36, 37]. The initial regionalization of SMPs during larval shell development could enable adult mantle modularity, and modularity has been hypothesized to drive novel shell features [32]. One could imagine that if an SMP responsible for prismatic-layer biomineralization is expressed in a new region within the shell field, that could result in a novel shell microstructure, promoting shell diversification [32]. To further explore such possibilities, we need to account for how the evolutionary age of an SMP relates to its expression domain within the shell field or mantle. For example, if lineage-restricted SMPs are responsible for specialized shell characteristics, perhaps they are localized to specific regions of the shell field. Likewise, a reasonable hypothesis is that conserved SMPs, which contribute to fundamental processes of biomineralization (e.g. calcium-regulation), may be more broadly expressed in shell field cells compared to lineage-restricted SMPs.

To understand how conserved vs. lineage-restricted SMPs might contribute to shell construction and diversification, accurate estimates of the evolutionary ages of SMPs are necessary. However, a finding from multiple proteomic comparisons of SMPs using BLAST approaches is that homologs could not be identified between the shell proteomes of different species [34, 38, 39]. One main conclusion stemming from the lack of homology in BLAST hits was that SMPs were deemed highly “lineage-specific”, meaning they were found only within a particular clade [36, 40, 41]. In many cases, the phylogenetic level to which SMPs are restricted remains unclear due to the small sample sizes of available shell proteomes relative to the number of representative species [12]. Additionally, previous comparative studies relied heavily on BLAST approaches, which have been shown to generate false-negatives for sequences that are short and repetitive (for which many SMPs are susceptible), resulting in an underestimate of orthologs between different species [42]. With the emergence of more molluscan shell proteomes, transcriptomes, and genomes we can now use new orthology inference approaches to identify orthologs with more confidence [9, 10, 19, 31, 38, 43–46]. These new approaches (e.g. OrthoFinder) [46] compensate for gene-length bias, and allow us to more confidently determine the evolutionary age of SMPs and hypothesize how SMPs influence shell morphology.

Here we present a toolkit of resources for studying molluscan SMP biology in two *Crepidula* species that are amenable to gene perturbation techniques (Fig. 1C and D). In particular we focus on *C. atrasolea* (a congener of the established model *C. fornicata)* that is an excellent species for studying shell development due to its availability year round in laboratory culture [20, 22]. We use our previous proteomic and transcriptomic work from *C. fornicata* [19] to identify the evolutionary age of its adult SMP repertoire and identify SMPs that are shared at different taxonomic levels with molluscs and metazoans. We then adapted in situ hybridization chain reaction (HCR) to *Crepidula* spp for the first time to examine the spatiotemporal expression patterns of a subset of SMPs during larval stages. With these data we revisit how SMP diversity and deployment during development might control shell evolution.

## Methods

### Animal culture

*C. atrasolea* [47] adults were maintained in the Lyons lab at the Scripps Institution of Oceanography, San Diego, California, USA. The animals were kept at 27 °C, in 100 mm x 20 mm plastic petri dishes (Corning # 430,167) with daily filtered natural seawater changes, and fed daily with 0.002% Phyto-Feast (Reed Mariculture Inc., Campbell, CA). Clutches of eggs inside their capsules were collected from the adults using forceps, as previously described in Henry et al. 2017, and placed in 35 mm x 10 mm plastic petri dishes (Falcon # 351,007) with bottle top-filtered (0.2 μm pore size; Thermo Scientific # 291–4520) natural sea water with streptomycin (2ug/mL; Sigma # S6501-100G) and penicillin (0.6 ug/mL; Sigma # 13752-5G-F) [20]. The collected embryos were then raised at room temperature (∼ 20 °C) inside their capsules until reaching the appropriate stages needed for fixation. Since *C. atrasolea* eggs are fertilized internally, the developmental age (days or hours post fertilization) of embryos was approximated based on hours or days since the embryo was uncleaved. The development of embryos within a single clutch is asynchronous, resulting in uncleaved, 2-cell, and 4-cell embryos all in the same clutch. In addition to days post fertilization (dpf), older embryos were also staged based on the morphology of their developing mouth, velar lobes, foot rudiment, and shell field. When embryos reached a desired stage, they were decapsulated under a dissecting scope using forceps in a gelatin coated 35 mm x 10 mm plastic petri dish prior to fixation or RNA extraction.

### Embryo fixation and fluorescent staining for shell development characterization

For embryos with ciliated velar lobes (starting at the organogenesis stage; Fig. 1C), individuals were relaxed in 7.5% magnesium chloride (dissolved in filtered natural seawater; Sigma-Aldrich #M7304) at benchtop for 30 min prior to fixation. Decapsulated embryos were fixed at ∼ 20 °C with 4% formaldehyde (Thermo Scientific # 28,908) in double filtered sea water for 60 min on a shaking plate followed by three 1 min 1X PBS Tween (1X Phosphate Buffered Saline: Gibco #70011-044, 0.5% Tween 20: Sigma-Aldrich P1379-100 mL) washes followed by three 10 min 1X PBS Tween washes. Animals were then dehydrated in methanol with three 5 min 100% methanol washes followed by three 1 min 100% methanol washes. Fixed individuals were stored at -80 °C in 100% methanol.

To visualize glycoproteins in the shell matrix, embryos were stained with wheat germ agglutinin (WGA), a lectin with a high affinity to N-acetylglucosamine and N‐acetylneuraminic acid residues [48–50]. Selected samples fixed in methanol (detailed above) were rehydrated into 5X SSCT solution (5X sodium chloride sodium citrate: Invitrogen # 15,557,044, 0.1% Tween 20) following 5 min 75% methanol 25% 5X SSCT, 50% methanol 50% 5X SSCT, 25% methanol 75% 5X SSCT, and two 100% 5X SSCT washes. Embryos were then stained in the dark with 0.001 mg/ml WGA (Alexa Fluor™ 594 Conjugate; Invitrogen W11262) and counterstained with 0.001 mg/ml hoechst in 5X SSCT for either 2–3 h at 20 °C or overnight at 4 °C overnight. Embryos were then rinsed with the 5X SSCT solution three times for 5 min at 20 °C. Samples were stored in the dark at 4 °C, and imaged within a week. Imaging methods are detailed below.

### RNA extraction and long read RNA sequencing

Three embryonic samples (Samples 1–3) spanning zygote through early juvenile development, and three samples (Sample 4–6) of *C. atrasolea* adults or their organs were collected for RNA extraction. Each embryonic RNA sample was made from two separate pools of embryos where RNA extractions were performed separately. Sample 1 contains one pool (*n* = 279) of uncleaved zygotes (0–8 hpf), 25-cell (24 hpf), and compaction (∼ 36 hpf) staged embryos; and a second pool (*n* = 186) of rectangular stage embryos (5 dpf). Sample 2 includes one pool (*n* = 180) of early organogenesis stage embryos (7 dpf) and a second pool (*n* = 168) of late organogenesis stage embryos (8–11 dpf). Sample 3 includes one pool (*n* = 133) of pre-hatch juveniles (14 dpf) and a second pool (*n* = 55) of post-hatched juveniles (14–15 dpf). Pools of embryos were kept separate and transferred to 1.5 mL tubes for RNA extraction. RNA from the separate pools of embryos were combined into the three samples as described above then sent for sequencing. Three samples (Sample 4–6) of *C. atrasolea* adults or their organs were collected for RNA extraction. Sample 4 contains one entire adult male specimen of *C. atrasolea.* Samples 5–6 contain RNA extracted from individually dissected organs from a single adult female. Sample 5 contains RNA only extracted from the mantle. Sample 6 contains RNA from Sample 5 along with RNA extracted from the remaining organs: head, goot, gill, visceral mass. Each dissected organ was kept separate for RNA extraction (supplementary methods S.1, Supplementary Material), and later recombined in equal parts to form Sample 6 prior to library preparation (supplementary methods S.2.1, Supplementary Material).

Total RNA was extracted using TRIzol (ThermoFisher), according to the manufacturer instructions (Invitrogen Doc. Part No. 15,596,026.PPS). Briefly, TRIzol was added to the collection tubes, and tubes were immediately frozen by placing them into a dry ice ethanol bath (70% ethanol, dry ice). Once frozen, mortar and pestle were used to completely homogenize tissues followed by the addition of chloroform to achieve separation of RNA in the aqueous layer. Precipitation of RNA from the aqueous layer was performed using 100% isopropanol, and after centrifugation, the RNA pellet was cleaned of impurities using 70% ethanol followed by resuspension in nuclease free water. For more details on RNA quality and quantity please refer to supplementary methods S.2.1, Supplementary Material. Six RNA samples (described above) were sent to the Roy J. Carver Biotechnology Center at the University of Illinois at Urbana-Champaign where the library preparation and sequencing were performed. 300 ng of total RNA were converted to cDNA with the Iso-Seq Express Oligo Kit. cDNA was barcoded with the Barcoded Overhang Adaptor Kit 8 A, and converted into a library with the SMRTBell Express Template Prep kit 2.0 (Pacific Biosciences). The libraries were pooled in equal concentration and the pool was sequenced on 1 SMRTcell 8 M on a PacBio Sequel IIe using the CCS sequencing mode and a 30hs movie time.

### Hybrid transcriptome assembly and annotation

Both short read transcriptomes generated by Illumina sequencing and long read transcriptomes generated by Iso-seq were used to construct a high coverage transcriptome for *C. atrasolea* (supplementary methods S.3, Supplementary Material). The long read transcriptomes were obtained as described in the supplementary methods S.3.1. The short read transcriptomes were downloaded from the NCBI Sequence Read Archive under accession number (SRP114839) [20]. We used rnaSPAdes (version 3.15.4) [51] to construct the hybrid transcriptome with both the short read and the long read data. The resulting transcriptome contains 848,512 transcripts, with a median length of 631 nt. CD-HIT (version 4.8.1) [52] was then used to merge redundant transcripts, resulting in 699,466 transcripts. Transcripts from non-eukaryotic species were then removed using alien index code [53], resulting in a transcriptome with 694,602 transcripts. The transcriptome was uploaded to TSA database where it underwent further adapter and contamination screening steps using FCS-GX (Astashyn et al., 2024). After foreign contamination was removed, the final accepted transcriptome uploaded to NCBI/TSA database contained 693,671 transcripts. We inferred a *C. atrasolea* proteome from this hybrid transcriptome using TransDecoder (version 5.5.0) [54] and used InterproScan (version 5.52) [55] to generate annotations for the inferred protein products. For additional details on the hybrid transcriptome and proteome assembly and annotation please see supplementary methods S.3.

To assess the quality of the transcriptome, BUSCO (version 5.3.2) [56] was used to score its completeness against Metazoa as well as Mollusca databases (metazoa_odb10 and mollusca_odb10). The hybrid transcriptome scored high completeness against both databases, with 99.2% for Metazoa (S:24.9%, D:74.3%, F:0.5%, M:0.3%) and 91.7% for Mollusca (S:27.0%, D:64.7, F:1.9%, M:6.4%).

### Identification of *C. atrasolea* SMPs in different IsoSeq libraries

The long read IsoSeq data consist of 6 individual samples including samples of adult tissue and samples spanning development (for more information see “RNA extraction and Long read RNA sequencing” above). To determine how many of the *C. atrasolea* SMP orthogroups are found in both embryonic development and adult tissues, we compared SMP orthogroup sequences with the raw IsoSeq data from each of the individual samples. We used Blast + command line to blast (tblastn) the sequences found in each SMP orthogroup to the individual IsoSeq sample sequences. If any of the sequences comprising the SMP ortholog had a blast hit to any of the individual samples, it was included as present in that sample.

### Species proteomes and data curation for orthology inference

In total 95 metazoan species proteomes were obtained from both publicly available databases and from a previously computed Orthofinder run (supplementary file S.1, Supplementary Materials) [43]. The 95 proteomes included in the Orthofinder run span 16 different metazoan phyla (including 61 molluscan species) and represent multiple sub-phylum-level metazoan clades. Additionally, 15 biomineral proteomes, which were for species whose proteomes were already included in the 95 proteomes (supplementary file S.1, Supplementary Materials), were analyzed using Orthofinder.

### Orthofinder2 analysis to identify orthogroups

To determine whether SMPs had orthologous copies in other species, orthology inference was used to identify conserved orthogroups within the previously mentioned input species proteomes using Orthofinder2 [46]. First, an all-vs-all DIAMOND (version 0.9.15) [57] search provided pairwise similarity scores for all sequences, and was used by Orthofinder2 to normalize for gene-length-bias and perform clustering of sequences into orthogroups using the MCL clustering algorithm [58]. Genes contained in each orthogroup are considered orthologs [46]. Second, sequences found within orthogroups were aligned using MAFTT (version 7.221) [59], and gene tree inference was performed using FastTree [60] to generate gene trees from multiple sequence alignments (supplementary figures S1: S13, Supplementary Materials).

### Kinfin analysis to identify lineage-restricted orthogroups

Orthogroups for each of the previously identified 185 *C. fornicata* SMPs were produced by Orthofinder2 and analyzed by Kinfin (version 1.0.3) [61] to identify lineage-restricted orthogroups at the species (*C. fornicata*), genus (*Crepidula*), family (Calyptraeidae), order (Littorinimorpha), class (Gastropoda), and phylum (Mollusca) levels. Based on these classifications, and at various times referred to in the text, the term “metazoan orthogroup” is used to refer to orthogroups that have molluscan *and* non-molluscan taxa represented. It is noted that non- metazoan species (e.g. eukaryotic outgroups) were not included in the Kinfin and Orthofinder analysis. Thus, the “metazoan orthogroups” likely contain many genes that are inherited from a more distant common ancestor to eukaryotes and metazoans. The broader term “molluscan orthogroup” is used to refer to orthogroups that contain only molluscan taxa. The term “lineage-restricted” is used to refer to orthogroups that contain proteins from more than one species that form a respective monophyletic clade; an exception being for a species-restricted gene (genes that were not found in any orthogroup).

### SMPs selected for in situ hybridization chain reaction (HCR)

HCR was used to examine the expression of SMPs from different lineage-restricted levels in *C. atrasolea* during mid organogenesis and veliger stages, prioritizing SMPs that were previously found differentially expressed in the mantle compared to the head, foot, and gill tissues of *C. fornicata* [19]. In total, the in situ expression patterns of 18 SMPs in 13 orthogroups were examined by HCR in *C. atrasolea* (supplementary file S.2, Supplementary Materials). These orthogroups contain 9 metazoan-restricted, 4 molluscan-restricted, and 5 gastropod-restricted SMPs. A *C. fornicata-restricted* SMP, *CfSMP6*, from orthogroup OG0088976 was also selected for HCR analysis. To annotate orthogroups, BLAST searches for all proteins within an orthogroup were performed against GenBank and their best reciprocal BLAST hit descriptions for all proteins were considered to arrive at a putative annotation for the orthogroup. As a result, 4 orthogroups returned BLAST hit descriptions of uncharacterized proteins, while the remaining 9 orthogroups returned descriptions of proteins that have been previously implicated in biomineralization including chitin-binding, calmodulin, lectin-like, and cysteine-protease (supplementary file S.1; supplementary file S.2, Supplementary Materials).

### In situ HCR

Following the HCR probe design protocol described in Kuehn et al. (2022), a custom software (ÖzpolatLab-HCR, 2021) was used to generate 15–30 DNA oligo probe pairs to *C. atrasolea* messenger RNA (mRNA) for each of the 18 genes of interest (supplementary file S.2, Supplementary Materials). We used our newly generated *C. atrasolea* transcriptome to design DNA probe pairs for our genes of interest. Using these DNA probe sequences, custom DNA oligos pools (50 pmol DNA oPools Oligo Pool) were ordered from Integrated DNA Technologies (Coralville, Iowa, USA). Probes were stored in nuclease-free and RNase-free H_2_O at a concentration of 1 pmol at -80 °C.

The HCR protocol was based upon those previously published for whole embryos in Kuehn et al. (2021) and Choi et al. (2018) [62, 63], with modifications to better suit our samples. Methanol fixed embryos were re-hydrated into 5X SSCT buffer before a 30 min pre-hybridization at 37 °C in probe hyb buffer (30% formamide: VWR # JT4028-1, 5X sodium chloride sodium citrate, 9 mM citric acid (pH 6.0): Sigma-Aldrich # C1909, 0.1% Tween 20, 50 µg/ml heparin: Sigma-Aldrich # H3393, 1X Denhardt’s solution: Thermo Scientific # AAJ63135AE, 10% dextran sulfate: Sigma-Aldrich # S4030). Embryos were then incubated in probe solution (1 pmol probe in probe hyb buffer) for 20–24 h at 37 °C. The probe solution was then removed with probe wash buffer (30% formamide, 5X sodium chloride sodium citrate, 9 mM citric acid (pH 6.0), 0.1% Tween 20, and 50 µg/ml heparin; at 37 °C) and 5X SSCT buffer (at 20 °C) rinses. Next, samples were incubated in the hairpin solution for 22–24 h at 20 °C. The following day, hairpins were removed with 5X SSCT buffer washes. Samples were then counterstained with 0.001 mg/ml hoechst in 5X SSCT (detailed above), and stored at 4 °C for up to a week. For additional details please refer to supplementary methods S.5, Supplementary Materials. Improved mRNA expression detection of *CaSMP1* in the shell field was observed using HCR vs. colorimetric in situ hybridization (supplementary fig. S14, Supplementary Materials), and thus HCR was the preferred method for examining the expression profiles of *C. atrasolea* SMPs.

Signal specificity was confirmed with control samples that were hybridized without a probe set. In the B3 546 fluorophore hairpin-only control veliger embryos, very faint labeling in the larval kidney, velum, and ocellus (supplementary fig. S15; fig. S16; fig. S17, Supplementary Materials) was noted. Faint background labeling of the larval kidney and ocellus was also noted in veliger staged B1 647 controls (supplementary fig. S15, Supplementary Materials).

### Mounting and imaging

Following Hoechst and/or WGA staining, individual specimens were mounted in either 80% Glycerol (Promega H5433) 20% 5X SSCT or 2% methyl cellulose in 5X SSCT (for posterior imaging) on Rain-X-coated (ITW Global Brands, Houston, TX) 22 × 50 mm glass coverslips for fluorescent imaging on a Zeiss LSM 700 on the 20x objective with an AxioCam HRm camera. Fluorescent Z-stack images were acquired in ZenBlack (Zeiss), then processed in ImageJ and Adobe Photoshop (Adobe Inc., California, USA). Samples for dark field images were mounted as described above, and imaged on a Zeiss Axio Imager M2 on the 10x objective with an AxioCam 506 Color camera. Darkfield Z-stack images were acquired in ZenBlue (Zeiss), Z-stacks were then flattened and focused in Helicon Focus (HeliconSoft (RRID: SCR_014462), and cropped in ImageJ. Figures were created in Adobe Illustrator (Adobe Inc., California, USA).

## Results

### Characterization and timing of shell development in *Crepidula atrasolea*

**Fig. 2:**
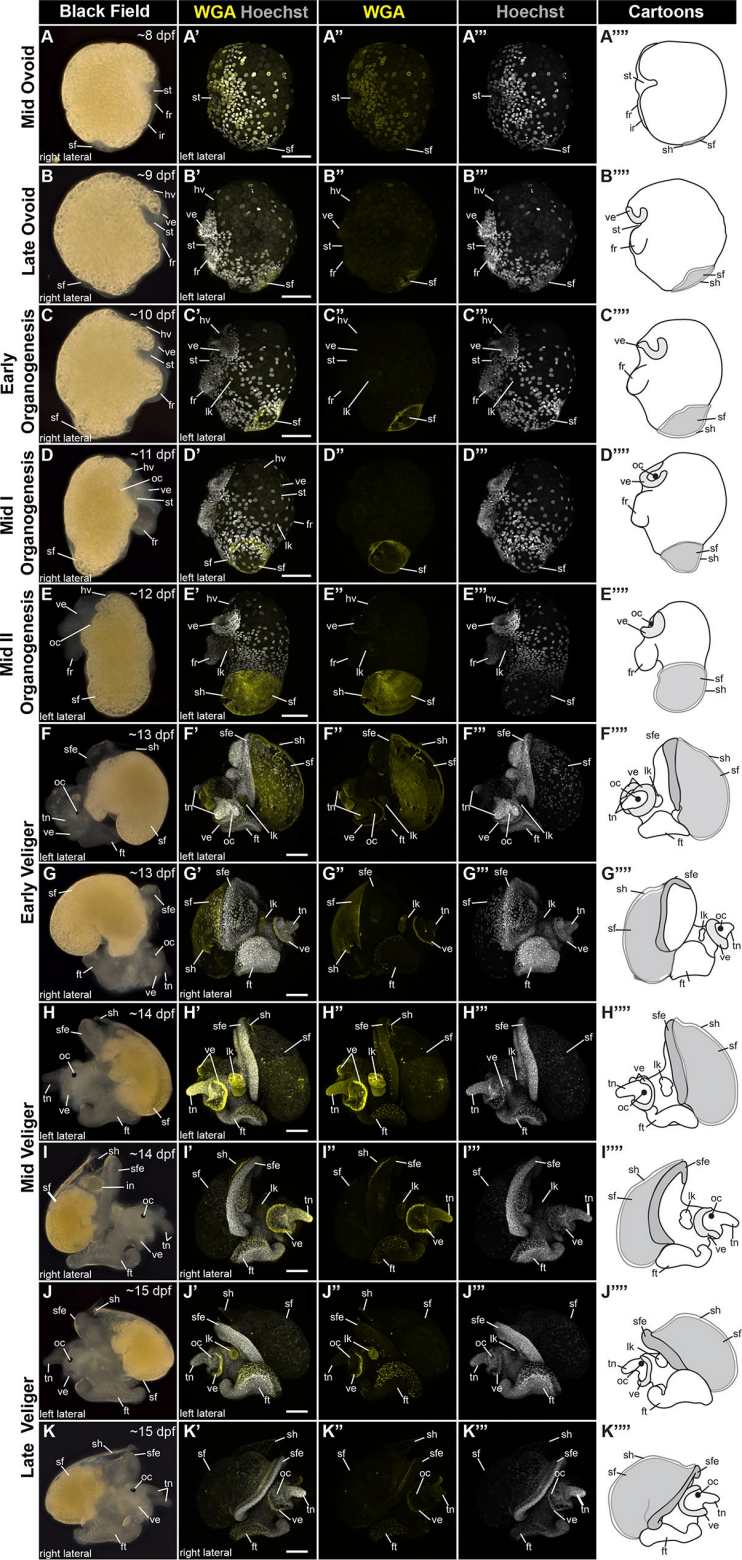
Characterization and timing of shell development in *Crepidula atrasolea.* Dark field and confocal images of fixed *C. atrasolea* embryos from approximately 8 days post-fertilization (dpf) to approximately 15 dpf at 20 °C. Fixed embryos were stained with wheat germ agglutinin (WGA) and imaged at the 20x objective on a confocal microscope. Cartoons highlight changes in shell morphology during development. In mid ovoid staged embryos (**A**), WGA stains nuclear membranes throughout the embryo. WGA begins to mark the shell field starting at the late ovoid stage (**B**). WGA continues to mark the developing shell throughout the organogenesis stages (**C-E**). WGA also marks the velar lobes, larval kidney, and foot in veliger staged embryos (**F-K**) Hoechst is shown in gray and WGA in yellow. fr, foot rudiment; ft, foot; hv, head vesicle; lk, larval kidney; in, intestine; ir, intestinal rudiment; oc, ocellus; sf, shell field; sfe, shell field edge; st, stomodeum; tn, tentacles; ve, velar lobes. Scale bars each represent 100 μm

To establish *C. atrasolea* as a model research organism for shell development in gastropods, we generated a detailed staging scheme examining stage-specific morphological features of shell development (Fig. 2). Refining the published staging scheme in [20], we based our stages on development at 20 °C with a focus on morphological changes to the shell field, the embryonic and larval shell-forming tissue. We found that wheat germ agglutinin (WGA), traditionally used to label cell membrane glycoproteins, marks the shell field and secreted shell material throughout larval development.

During larval development, the dorsal epithelium invaginates to produce a transient structure, known as the shell gland [23, 64], where organic shell secretions are first detected [23, 65]. The shell gland tissue evaginates to form the shell field. We first detected organic shell secretions–as assayed by WGA staining–in the shell field during ovoid stages (8–9 dpf). Larval structures, including the foot rudiment, stomodeum, shell field, and intestinal rudiment, were first visible in mid ovoid embryos at ∼ 8 dpf, when elongation along the anterior-posterior axis is apparent (Fig. 2A). During the mid ovoid stage, the posteriorly-located shell field is translucent and rounded. Translucent ventral structures, including the velar lobes and foot rudiment, become more prominent. At the mid ovoid stage, WGA intensely stains the outlines of cell nuclei throughout the embryo and very weakly marks the developing shell field in the posterior terminus (Fig. 2A’’). The late ovoid stage (∼ 9 dpf) is distinguished by the presence of two ciliated velar lobes, a lengthened foot rudiment, and a flattened shell field along the dorsal-posterior end of the embryo (Fig. 2B). At this stage, glycoprotein secretion from the shell field cells into the extracellular matrix is first strongly visible, as indicated by bright WGA staining (Fig. 2B’).

During organogenesis stages (10–12 dpf), larval shell accretion corresponds to elongation along the anterior-posterior axis (Fig. 2C: Fig. 2E). In the early organogenesis stage (∼ 10 dpf), the shell field begins to diverge from the midline for the first time, as it rounds out in the posterior of the embryo (Fig. 2C). WGA staining is visible in the larval shell secretions and larval kidney during early organogenesis (Fig. 2C’’). At the mid I organogenesis stage (∼ 11 dpf), the shell field elongates along the anterior-posterior axis, larval organs continue to develop, and pigmented ocelli are visible (Fig. 2D). The larval shell field thickens and expands in the mid II organogenesis stage (12 dpf), and the pigmented ocelli are pronounced (Fig. 2E). Increased WGA staining highlights shell field expansion during mid I organogenesis (Fig. 2D’) and mid II organogenesis (Fig. 2E’).

In veliger stages (∼ 13 dpf to ∼ 20 dpf), the larval shell is fully formed, and larval organs are well developed (Fig. 2F: Fig. 2K). At the early veliger stage, an enlarged foot extending ventrally along the body, enhanced velar lobes, and paired tentacles are also present (Fig. 2F and G). The WGA staining of secreted shell materials (e.g. glycoproteins) diminished during the veliger stages. At mid veliger stages (∼ 14 dpf) the shell is angled upwards (Fig. 2H and I), and then flattens out to cover the entire animal during late veliger stages (Fig. 2J and K). During the veliger stages, the yolk reserves begin to diminish and are no longer visible in the hatched juvenile (∼ 21 dpf).

### Most SMPs have conserved orthologs with only a few being species-restricted to *C. fornicata*

**Fig. 3:**
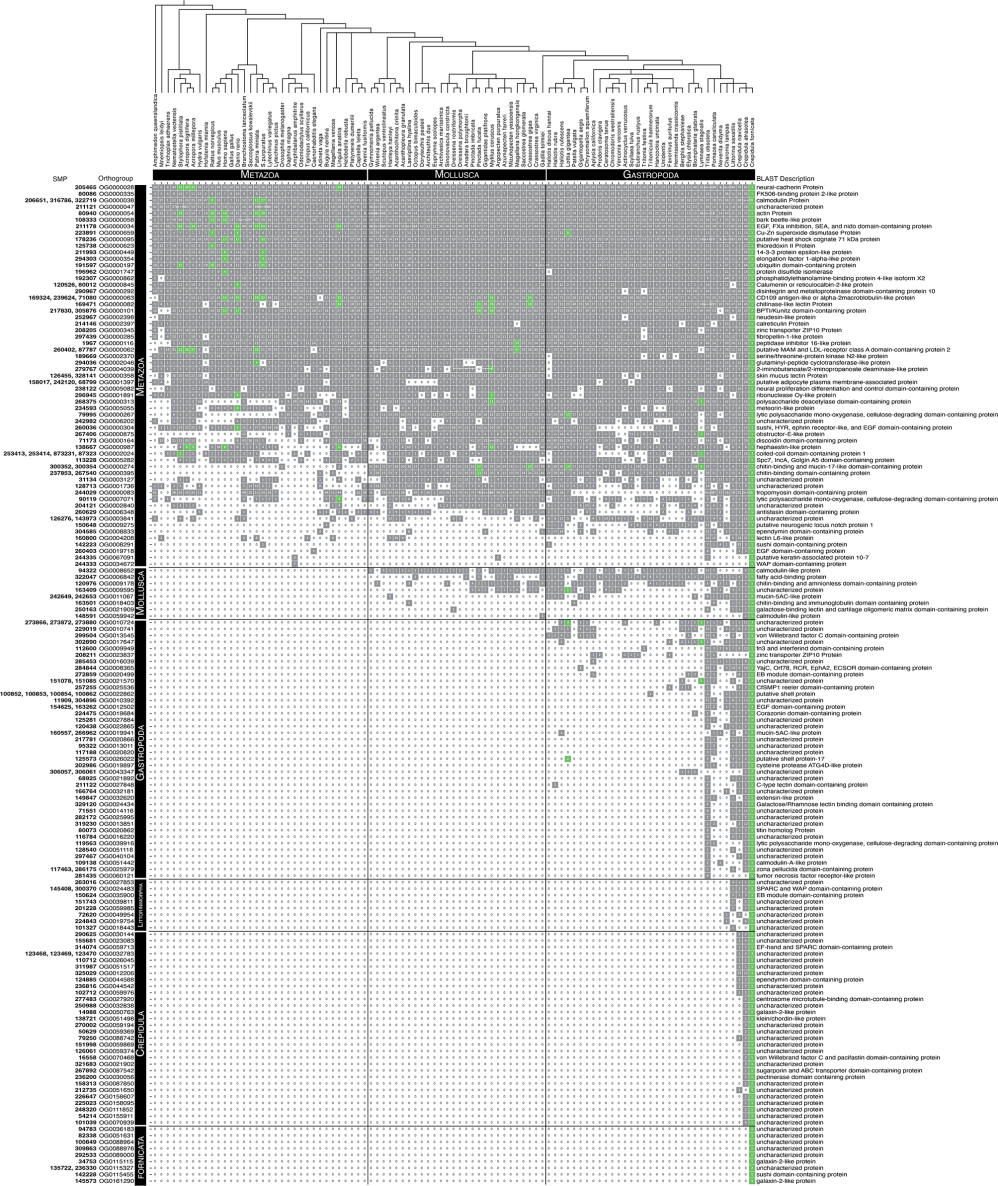
Shell matrix proteins of *Crepidula fornicata* clustered into orthogroups. Rows represent SMP orthogroups that were generated through ortholog inference techniques, and ordered based on the taxonomic level to which they are found to be lineage-restricted. Columns depict counts of proteins per taxon with their order (tree depicted above columns) based on current understandings of their phylogenetic positions (Kocot et al. 2011; Laumer et al. 2015; Laumer et al. 2018; Cunha and Giribet 2019). Gray boxes indicate presence of at least one protein from a taxon in an orthogroup. White boxes indicate that no protein was found for a respective taxon in an orthogroup. Green boxes indicate the presence of a skeletal matrix protein in the given orthogroup

**Fig. 4:**
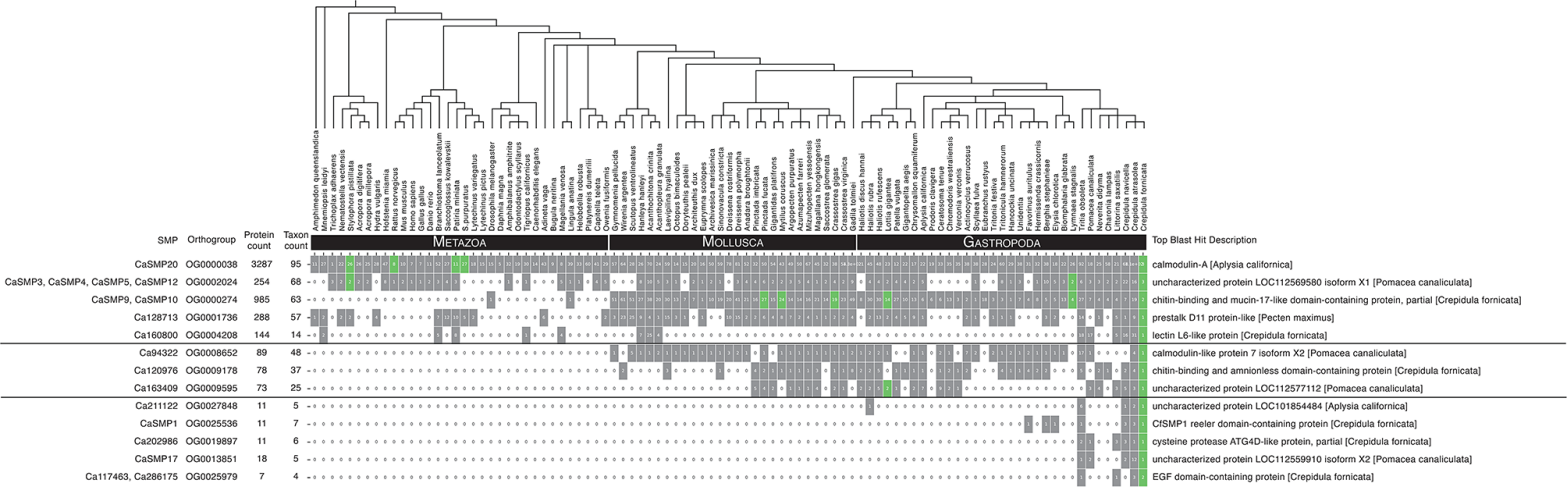
Lineage-restricted shell matrix proteins selected for HCR in *Crepidula atrasolea*. Rows represent SMP orthogroups that were examined by HCR in *C. atrasolea.* They are ordered based on the taxonomic level to which they are found to be lineage-restricted (*Metazoa*-restricted, *Gastropoda*-restricted, and *Crepidula*-restricted). Columns depict counts of proteins per taxon with their order (tree depicted above columns) based on current understandings of their phylogenetic positions (Kocot et al. 2011; Laumer et al. 2015; Laumer et al. 2018; Cunha and Giribet 2019). Gray and white boxes indicate presence or absence of respective species within SMP orthogroups. Green boxes indicate the presence of a skeletal matrix protein in the given orthogroup

We asked whether *C. fornicata* SMPs have orthologs in other species, or whether most are species-restricted SMPs. Furthermore, we asked whether or not we could determine the evolutionary age of those SMPs through an orthology inference approach. To identify orthologs of *C. fornicata* in *C. atrasolea*, we used our newly assembled hybrid *C. atrasolea* transcriptome (described in methods above). Using the orthology inference program Orthofinder, we examined the proteomes of 95 metazoan species to determine the level at which 185 SMPs from *C. fornicata* are restricted to. In total, Orthorfinder assigned 89.1% (*n* =  3,777,255 of 4,237,370) of the input proteins to 213,404 orthogroups. We examined the Orthofinder results and determined that 95% (*n* = 175 of 185) of the SMPs were assigned to 145 orthogroups containing two or more species (Fig. 3; Fig. 4). In total, we found that 40% (74 of 185) of SMPs were in orthogroups that contain taxa from at least one molluscan species and one non-molluscan species, which we classify as a “metazoan orthogroup” (see Methods). Additionally, we determined that the remaining 55% (101 of 185) of SMPs are found within orthogroups that contained only molluscan taxa, which we classify as a “molluscan orthogroup”.

These molluscan orthogroups were further sub-divided into multiple lineage-restricted SMPs: 5% (*n* = 9 of 185) Molluscan-restricted; 27% (*n* = 51) Gastropod-restricted; 5% (*n* = 9) Littorinimorpha-restricted; 17% (*n* = 32) *Crepidula*-restricted (Fig. 3). We observed that *Crepidula*-restricted SMP orthogroups were responsible for nearly one sixth of all SMP orthogroups, and that of the two other *Crepidula* species in the dataset, *C. atrasolea* had orthologs for 91% (*n* = 168 of 185) of all SMPs, compared to 72% (*n* = 134 of 185) for *C. navicella.* Interestingly, in the IsoSeq data the majority of *C. fornicata* SMPs conserved in *C. atrasolea* (116/167) were found in both larval and adult stages (supplementary file S3, Supplementary Materials). We note that fewer *C. atrasolea* SMP orthologs (167 vs. 168) were detected using only IsoSeq data compared to the Orthofinder findings, likely due to the combined transcriptome being more complete. Our results showed that only 5% (10 of 185) of SMPs were not found in any other species, suggesting that few species-restricted SMPs exist in the shell proteome. Taken together, these results demonstrate that the majority of SMPs have conserved orthologs while few SMPs are actually novel to *C. fornicata*.

### Shared shell field-specific expression of *SMP1* in *C. atrasolea* and *C. fornicata* embryos

**Fig. 5:**
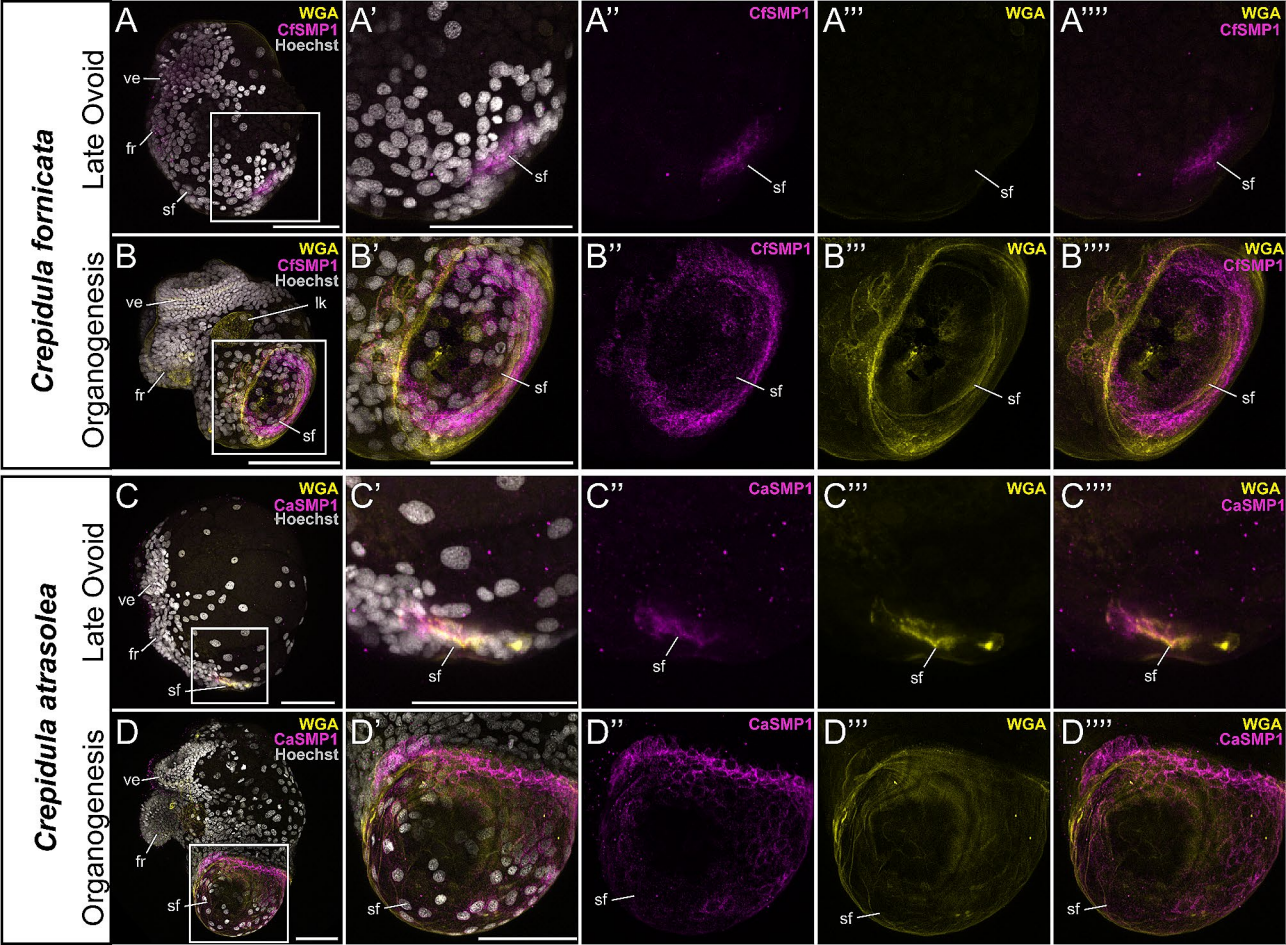
Shell field expression of Shell Matrix Protein 1 in *Crepidula fornicata* and *Crepidula atrasolea* embryos. Using hybridization chain reaction (HCR), mRNA expression was detected for SMP1 in both species (**A-D**). Wheat germ agglutinin (WGA) was used to mark the larval shell. *CfSMP1* is expressed throughout the shell field in late ovoid staged *C. fornicata* embryos (**A**), and more intensely around the shell field edge during organogenesis (**B**). Similar expression patterns are seen for CaSMP1 in late ovoid staged (**C**) and organogenesis staged *C. atrasolea* embryos (**D**). Hoechst is shown in gray, SMP1 in magenta, and WGA in yellow. fr, foot rudiment; ft, sf, shell field; ve, velar lobes; lk, larval kidney. Scale bars represent 100 μm

To confirm that gastropod-restricted SMPs have comparable expression patterns in *C. fornicata* and *C. atrasolea*, we compared larval expression of Shell Matrix Protein 1 (*SMP1*), the most upregulated SMP in the adult *C. fornicata* mantle [19], in the two species. The species comparison of *SMP1* also demonstrated that we can apply previous proteomic and transcriptomic findings from *C. fornicata* to *C. atrasolea*. We found exclusive shell field expression of *SMP1* in both *C. fornicata* and *C. atrasolea* (Fig. 5). The *CaSMP1* homolog we found contains an extracellular matrix-binding reeler domain, which is also found in *C. fornicata*. Based on the morphology of the velar lobes and foot rudiment, we identified late ovoid and late organogenesis stage embryos as the most comparable stages between the two species (Fig. 1C). Using HCR, we found that expression of *CfSMP1* and *CaSMP1* was confined to cells within the shell field at the ovoid stages (Fig. 5A and C), and a stronger ring of expression was seen in the shell field edge during organogenesis (Fig. 5B and D). We used WGA to stain the larval shell field in *C. fornicata and C. atrasolea* embryos (Fig. 5B: Fig. 5D). WGA staining of the shell field in *C. fornicata* was very faint at the ovoid stage (Fig. 5A’’’) and most prominent at the organogenesis stage (Fig. 5B’’’). Meanwhile, WGA staining was bright in both ovoid (Fig. 5C’’’) and organogenesis staged embryos of *C. atrasolea* (Fig. 5D’’’). In both species, the WGA staining encompasses the SMP1 expressing cells (Fig. 5A: Fig. 5D). Together, *SMP1* expression and WGA staining revealed a more dorsal-lateral positioned, and flatter, shell field in *C. fornicata* embryos compared to the larval shell field of *C. atrasolea*.

### 12 out of 18 SMPs examined exhibit regionalized expression in the larval *C. atrasolea* shell field

**Fig. 6:**
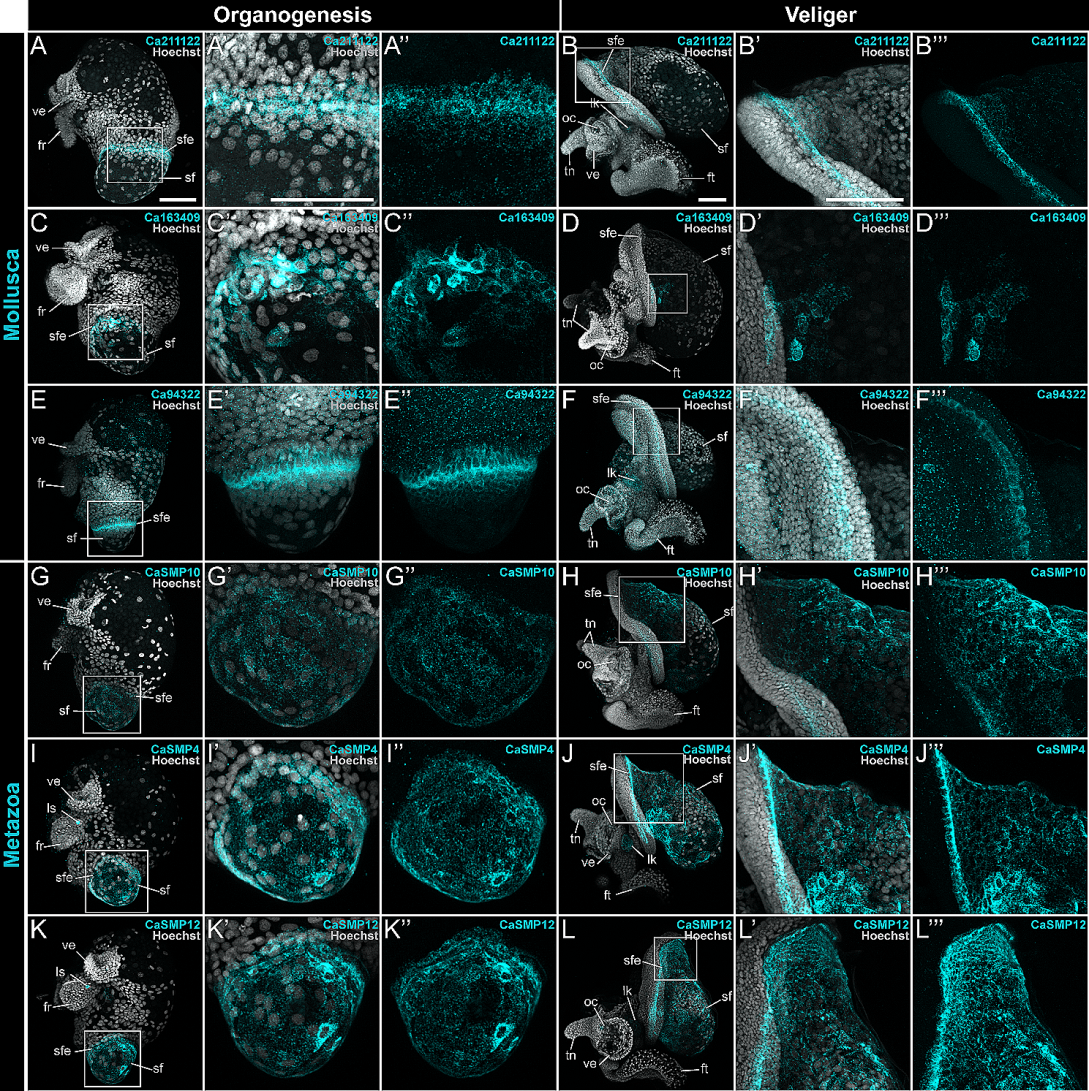
Shell field expression of SMPs in *Crepidula atrasolea* at organogenesis and veliger stages. Using hybridization chain reaction, mRNA expression was detected for *Ca211122* (**A** and **B**), *Ca163409* (**C** and **D**), *Ca94322* (**E** and **F**), *CaSMP10* (**G** and **H**), *CaSMP4* (**I** and **J**), and *CaSMP12* (**K** and **L**). Hoechst is shown in gray and each SMP in cyan. ft, foot; sf, shell field; sfe, shell field edge; tn, tentacles; ve, velar lobes; ls, larval statocyst; lk, larval kidney. Scale bars in each represent 100 μm

**Fig. 7:**
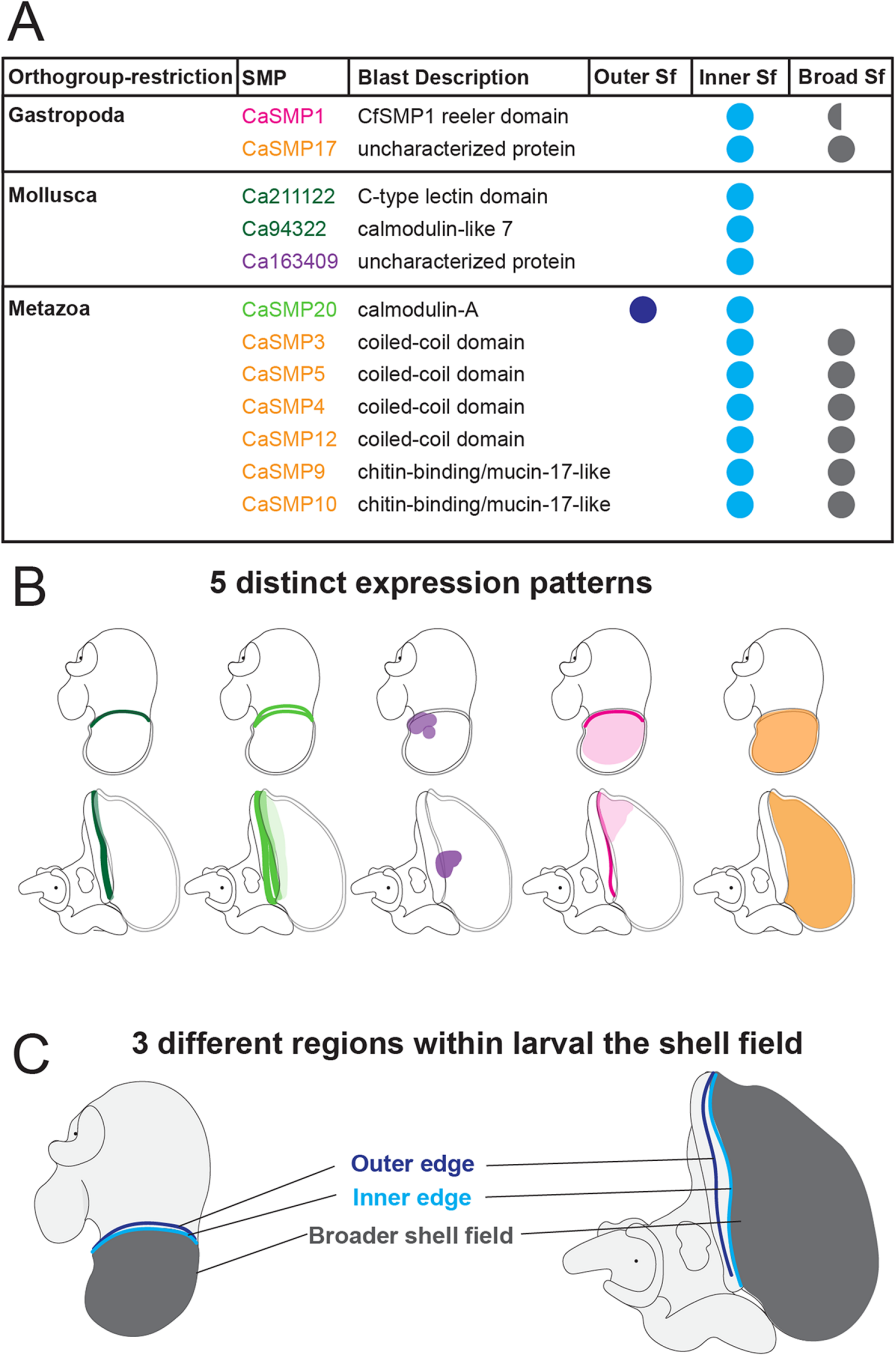
Summary of regionalized SMP expression during larval shell development in *Crepidula atrasolea*. Summary table showing the lineage-restriction of each SMP, the best blast hit description, and shell field region(s) of expression (A). Circles in (**A**) indicate presence or absence of expression in the outer edge (outer Sf), inner edge (inner Sf), and/or the broader shell field (broad Sf). Half circles indicate presence of expression in one stage (late organogenesis or veliger) but not both. Cartoons depicting 5 different SMP expression patterns within the shell field of organogenesis and veliger stage *C. atrasolea* embryos (**B**). The SMPs are color coded in (**A**) based on their expression patterns shown in (**B**). Cartoons showing regionalization of the shell field into 3 potential zones of biomineralization in late organogenesis and veliger stage *C. atrasolea* embryo (**C**). These zones are highlighted in different colors: outer shell field edge in dark blue, inner shell field edge in light blue, and broader shell field in dark gray (**C**)

To identify SMPs expressed in the shell field of *C. atrasolea*, we characterized the spatial-temporal expression of 18 different SMPs from 13 different orthologous groups at the late organogenesis and veliger stages. Out of the 18 genes we examined, 12 were expressed in the shell field, and five (*CaSMP1*, *CaSMP10*, *CaSMP9*, *CaSMP17*, and *Ca211122*) were exclusively expressed in the shell field (Fig. 6; supplementary fig. S18; supplementary file S.2, Supplementary Materials). We found six SMPs without shell field expression in *C. atrasolea* at the late organogenesis and veliger stages (supplementary fig. S19, Supplementary Materials). Many of the SMPs we examined were expressed in other tissues outside of the shell field, including in the statocyst (supplementary file S2, Supplementary Materials). Overall, we found two general SMP expression patterns in the shell field: SMPs with restricted expression in the shell field edge (Fig. 6A: Fig. 6F; supplementary fig. S18, Supplementary Materials), and SMPs with broader expression throughout the shell field (Fig. 6G: Fig. 6L; supplementary fig. S18, Supplementary Materials). Within these general domains, we found 5 distinct expression patterns (Fig. 7A and B). Based on the spatial-temporal location of these expression patterns, we identified three primary regions of the shell field: the outer edge, the inner edge, and the broader shell field (Fig. 7A and C).

#### Restricted shell field edge expression of uncharacterized molluscan and gastropod-specific SMPs

We found restricted shell field edge expression of *Ca211122*, a gastropod-restricted c-type lectin domain-containing SMP, in *C. atrasolea* (Figs. 4 and 6A and B). In late organogenesis stage embryos, *Ca21122* expression was strongest in a continuous band of cells along the shell field edge (Fig. 6A). During the veliger stages, *Ca211122* persisted in the shell field edge and was weakly expressed in the dorsal-anterior portion of the shell field (Fig. 6B). We found another mollusc-specific uncharacterized SMP, *Ca163409*, with restricted expression in the shell field edge (Fig. 6C and D). In mid organogenesis stage embryos, the uncharacterized molluscan SMP, *Ca163409*, was expressed in a subset of ventral-laterally positioned shell field cells (Fig. 6C’). The most prominent expression was detected adjacent to the shell field edge (Fig. 6C’’). In veliger stage embryos, *Ca163409* was expressed in a small discrete group of cells located within the anterior portion of the shell field, adjacent to the shell field edge (Fig. 6D). We noted variability in the number of shell field cells labeled between specimens, and also noted anterior expression in the head of one individual (supplementary fig. S20, Supplementary Materials).

#### Restricted shell field edge expression of molluscan-specific calmodulin SMP

We identified a molluscan-specific calmodulin-like 7 gene, *Ca94322*, with strong expression in one ring of cells around the shell field edge of the larval shell field (Fig. 6E). *Ca94322* expression diminished in cells further from the shell field edge (Fig. 6E’’). At the veliger stage, the most intense expression was seen within a continuous band of *Ca94322*-positive cells between the outer and inner shell field edge (Fig. 6F and F’’). During the veliger stages, *Ca94322* expression expanded to the anterior portion of the foot, and diminished levels were detected throughout the head of the embryo (Fig. 6F). The shell field edge-specific patterns of *Ca94322* were similar to those reported in *P. fucata* [66] and *H. asinina* [67, 68]. These *P. fucata* and *H. asinina* calmodulin genes are not orthologs to *Ca94322*, but were instead placed in another calmodulin orthogroup with Calmodulin-A, *CaSMP20*.

#### Broad shell field expression of SMPs from metazoan orthogroups

Mucin and mucin-like genes are cell surface glycoproteins [69]. Mucins are secreted by epithelial cells and aid biomineralization of vertebrate bones, teeth, and cartilage [70]. Mucin-like genes have been previously identified in molluscan mantle tissue, and are hypothesized to aid shell biomineralization [71–73]. In *C. atrasolea*, we identified two mucin-like genes, *CaSMP10* (Fig. 6G: Fig. 6H) and *CaSMP9* (supplementary fig. S18, Supplementary Materials), in the metazoan orthogroup (Fig. 4). We found that these genes are expressed throughout shell field cells during organogenesis and veliger stages (Fig. 6G: Fig. 6H). While expression is distributed throughout the shell field, the brightest signal is detected in the dorsal-anterior cells of the veliger shell field (Fig. 6H’’).

We found four coiled-coil domain-containing SMPs (*CaSMP3*, *CaSMP4*, *CaSMP5*, and *CaSMP12*) in a metazoan orthogroup, with patchy expression throughout the shell field at both the late organogenesis and veliger stages (Figs. 6I and 6L: supplementary fig. S18, Supplementary Materials). In the late organogenesis stages, *CaSMP4* & *CaSMP12* expressing cells were also located in the statocyst (as seen in Fig. 6I and K). While there was some individual variability in the intensity of signal, we also observed bright expression in a band of squamous cells in the shell field edge during organogenesis (Fig. 6I’ and Fig. 6K’). In the veliger stages, patchy expression of these genes persisted throughout the shell field, with a brighter, continuous band of cells labeled in the shell field edge (Fig. 6J’’’; Fig. 6L’’’). *CaSMP4* positive cells were highly concentrated in an anterior-ventral patch within the shell field where the shell is curved to one side (Fig. 6J).

### Co-expression of *CaSMP3* and *CaSMP20* mRNA reveals three distinct shell field cell populations and confirms shell field regionalization during larval development

**Fig. 8:**
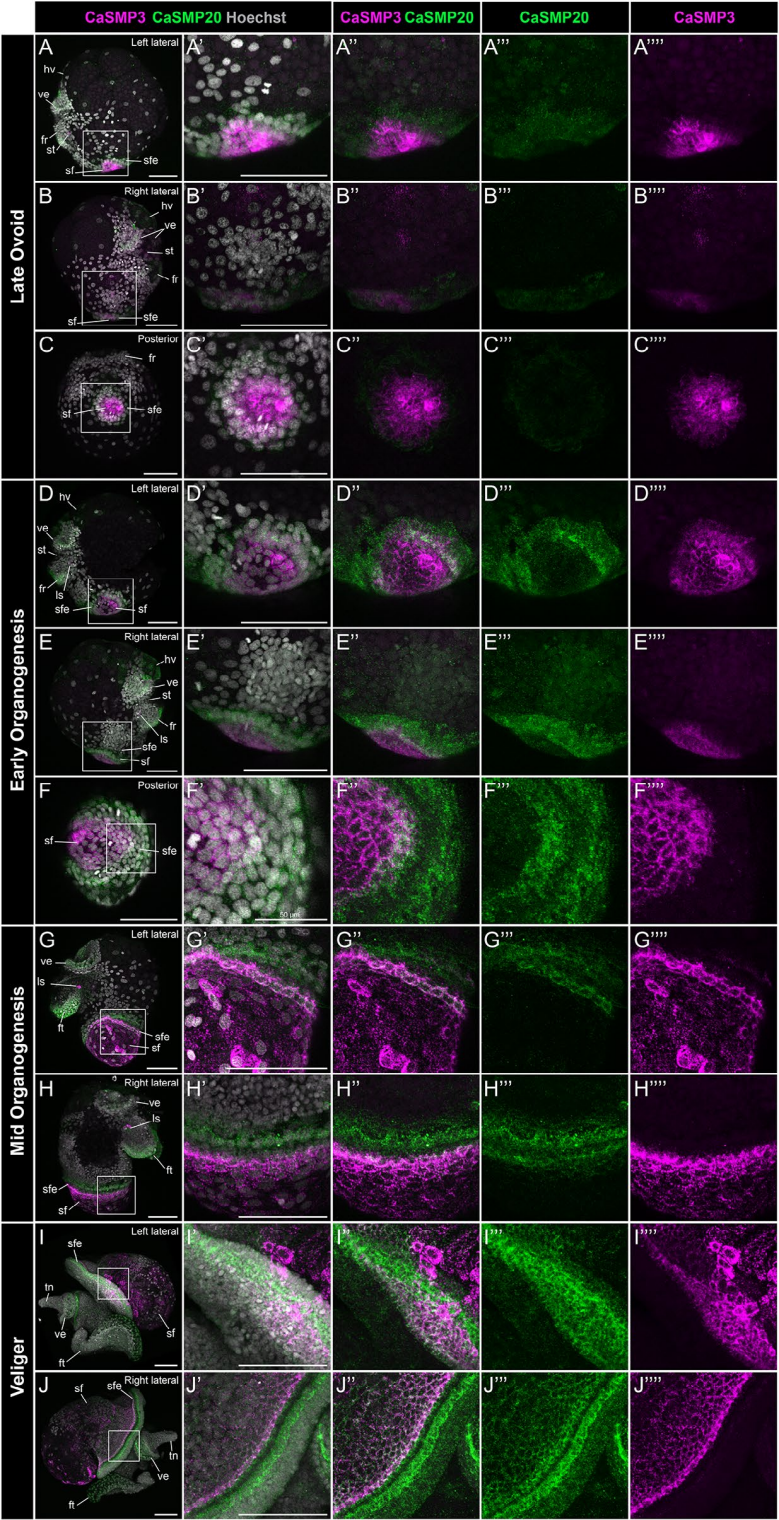
Co-expression of *CaSMP3* and *CaSMP20* mRNA throughout embryonic shell development in *Crepidula atrasolea*. Right lateral (**A**), left lateral (**B**), and posterior views (**C**) of late ovoid embryos show co-expression of *CaSMP3* (magenta) and *CaSMP20* (green) in the shell field. These two expression domains continue during early organogenesis (**D-F**) and mid organogenesis (**G-H**). In the veliger stages, laterally asymmetric expression is most apparent in the shell field edge (**I-J**). Hoechst is shown in gray, *CaSMP3* in magenta, and *CaSMP20* in green. ft, foot; sf, shell field; sfe, shell field edge; tn, tentacles; ve, velar lobes; ls, larval statocyst. Scale bars each represent 100 μm unless otherwise specified

To confirm that different regions of SMP expression (e.g. broader vs. shell field edge specific regions) correspond to distinct shell field cell populations, we examined the spatial-temporal expression of *CaSMP3* (broader shell field domain) and *CaSMP20* (shell field edge specific region) throughout larval shell development (Fig. 8). We confirmed that *CaSMP20* and *CaSMP3* have two discrete regions and one overlapping region of expression in late ovoid to veliger staged embryos (Fig. 8). We identified at least three shell field cell populations during larval development: a *CaSMP3* + population in the shell field, a *CaSMP20* + population lining the edge of the shell field, and a *CaSMP3*+/*CaSMP20* + population in between.

Specifically, at the late ovoid stage, posteriorly located CaSMP3 positive cells were surrounded by a fainter ring of *CaSMP20* expression, with a small population of cells expressing both *CaSMP3* and *CaSMP20* (Fig. 8A: Fig. 8C). At the early organogenesis stage three cell populations are discernible by a patch of CaSMP3 expression in posterior end, a ring of *CaSMP3* and *CaSMP20* co-expression around the shell field, and a second, outer ring of *CaSMP20* expression in the shell field edge (Fig. 8D: Fig. 8F). At this stage, these three cell populations were most visible on the posterior end (Fig. 8F’ and Fig. 8F’’). In mid organogenesis embryos, *CaSMP3* expressing cells were located throughout the shell field, *CaSMP3* and *CaSMP20* co-expressing cells were concentrated to a band along the shell field edge, and *CaSMP20* expressing cells were situated along a second band above the *CaSMP3* and *CaSMP20* positive cell population (Fig. 8G’ and Fig. 8H’). At the veliger stage, we saw a laterally asymmetric distribution of shell field cell populations (Fig. 8I and J). There was only one, thicker band of *CaSMP20* positive cells on the left lateral side (Fig. 8I’’’), with the *CaSMP3* and *CaSMP20* co-expressing cell population restricted to a smaller patch of the shell field edge (Fig. 8I’’). In contrast, on the right lateral side there were two prominent bands of *CaSMP20* expression within the shell field edge (Fig. 8J’’’). *CaSMP3* and *CaSMP20* were co-expressed in cells of the second, posterior-most band (Fig. 8J’’). The third cell population, marked by CaSMP3 expression, was located throughout the shell field (Fig. 8J’’’’).

We also observed larval expression of *CaSMP3* and *CaSMP20* outside of the shell field, with higher levels of *CaSMP20* expression in anterior and ventral tissues compared to CaSMP3 (Fig. 8). From the late ovoid to veliger stages, the *CaSMP20* signal was detected faintly in patches throughout the embryo and more prominently in three non-shell field structures: the posterior edge of the foot rudiment, the head vesicle, and the velar lobes (Fig. 8A: Fig. 8F). Apart from the shell field, *CaSMP3* was only expressed in the larval statocysts (Fig. 8A: Fig. 8H). Taken together, *CaSMP3* and *CaSMP20* exhibit co-expression in the shell field and mark SMP expressing cell populations during shell development in *C. atrasolea*. Given the different SMP expression patterns detected (Fig. 7B), there are at least three different cell populations in the larval shell field of *C. atrasolea*.

## Discussion

### *C. fornicata and C. atrasolea*: complementary models for studying molluscan biomineralization

In our previous study [19], we used the large-bodied species *C. fornicata* to extract peptides from its shell and perform differential gene expression analysis on different adult organs to determine which shell matrix proteins are highly expressed in the mantle. We also performed a preliminary in situ hybridization screen to determine which of these highly-expressed SMPs were expressed earlier in the larval shell field [19]. But to make a more comprehensive analysis of SMP expression in development–and to ultimately knock out specific SMPs in the future–we chose to switch to *C. atrasolea* [20, 22]. While *C. fornicata* is an indirect developer and has a long generation time (> year), *C. atrasolea* is a direct-developer with a generation time of just ∼ 4 months. Even a modest colony (several dozen mature reproductive females and an equal number of younger males) can provide embryos year-round in a recirculating sea water system [20, 22], making it the more practical species for day to day use.

We found comparable expression patterns of the gastropod-specific SMP1 gene in the shell field of both species, which suggests that previous finds in *C. fornicata* can be applied to work in *C. atrasolea*. We also found 10 SMPs that are expressed in the adult stage of *C. fornicata* [19], that are also expressed during larval shell development in *C. atrasolea*, suggesting shared SMP expression in the adult mantle and larval shell field. Leveraging data from both species, we provide a comprehensive analysis of larval SMPs and build a foundation of knowledge that can be used to design future studies. For example, these two species could be used to study what, if any, aspects of SMP expression are related to the direct versus indirect life cycle. Or, SMPs could be functionally perturbed, since both species are amenable to CRISPR/Cas9 genome editing [20–22].

### “Adult SMPs” are also expressed during larval shell development in *C. atrasolea*

Proteomic investigations using larval shells in bivalves revealed drastically different repertoires of SMPs during larval and adult shells. Zhao et al. (2018) identified only 4 SMPs that were shared between larval and adult bivalves; Carini et al. (2019) found 45 larval SMPs with only four sequences sharing similar domains with their adult complement; and Cavallo et al. (2022) identified only 1 out of 5 adult SMPs with larval expression in the Antarctic bivalve *Laternula elliptica*. By contrast, we found many SMPs that are expressed in adult and larval stages in *C. fornicata and C. atrasolea*. There are a few possible explanations for the discrepancy between our results and studies in bivalves. First, adult and larval biomineralization might be more similar in gastropods than they are in bivalves. Second, it might reflect the different methods used to identify SMPs: we have not sequenced larval shell field tissue or performed proteomics on the larval shell, thus missing larval-specific SMPs. More research dedicated to comparisons of larval and adult biomineralization will be very useful, especially if the same methods and analyses are applied to both stages, in the same species.

### Previous approaches underestimate evolutionary conservation of SMP orthogroups

Understanding the phylogenetic relationships of SMPs is essential for tracing the evolution of biomineralization in marine invertebrates. Within the past two decades, a surprising outcome from proteomic investigations of biomineral structures is the preponderance of lineage-restricted shell matrix proteins [74]. Examples in bivalves include 52% in *Pinctada maxima*, 54% in *Mya truncata*, and 66% *Mytilus edulis* of SMPs had no BLAST hit [75]. These observations have been informed in part by BLAST pairwise similarity *E*-value scores against large sequence databases, under the assumption that high pairwise similarity-scoring SMPs may have evolved more recently because they lack homologous reciprocal BLAST hits. Indeed, close examination of the sequences of lineage-restricted SMPs has shown that they contain long stretches of repetitive, low complexity regions [40], which are generally a hallmark of recently-diverging genes [76]. Likewise, in our previous study we took a BLAST approach and determined that 29% of *C. fornicata* SMPs had no best-reciprocal BLAST hit against sequences in GenBank, and that many of these SMPs contain long stretches of repetitive low complexity domains [19].

However, a lack of reciprocal BLAST hit is not always a reliable measure of lineage restriction or recent divergence events, as it can be an artifact due to homology detection failure in heuristic BLAST searches, which result in high numbers of false-negatives, especially in short and rapidly evolving genes [42]. This is due to the BLAST algorithm placing greater emphasis on pairwise-similarity scores that are directly correlated with gene-length and without regard to variable sequence evolution. This detection failure suggests that previously classified ‘species-restricted’ SMPs that were assessed through BLAST approaches could have homology outside of their own lineages, but due to their highly divergent sequences, their degree of similarity does not meet a certain threshold by BLAST standards. Reassessment of previous classifications using an orthology inference approach is needed to confirm these observations.

A second explanation for homology detection failure is the underrepresentation of species in GenBank. We observed a large number of *C. fornicata* SMPs are shared by two other *Crepidula* species: *C. navicella* and *C. atrasolea*. These two species were found to have orthologs for 91% (*C. atrasolea*) and 72% (*C. navicella*) of *C. fornicata* SMPs. Without these two species, the number of *fornicata*-restricted SMPs would increase from 5 to 23%, the latter being similar to our previous findings that 29% of *C. fornicata* SMPs had no BLAST hits. This discrepancy could be explained by underrepresented taxa, especially from related species, being absent in BLAST databases like GenBank. This leaves open the possibility that other underrepresented taxa, in addition to gene-length-bias, may result in homology detection failure. These results underscore the importance of broad taxon sampling, and data accessibility, for future studies of molluscan biomineralization genes. In the course of this study we constructed a new, comprehensive *C. atrasolea* transcriptome that should aid future work. Likewise, our Orthofinder dataset will be useful for studies of biomineralization in other molluscan systems.

### *Crepidula* SMPs have regionalized shell field expression

Molluscan mantle modularity has been widely observed in adult and juvenile tissue [32, 33, 36, 66, 77, 78]. By contrast fewer studies focused on larval tissue [24, 26, 65, 79]. During larval shell development of *Lymnaea stagnalis*, Herlitze et al. (2018) detected regionalized expression of SMPs to specific zones of the shell field. Similarly, in *C. atrasolea* we found distinct regions of SMP expression: broad expression throughout the shell field, and more restricted expression in the shell field edge. Through HCR and confocal imaging, we were able to attain a high cellular and subcellular resolution of the mRNA localization, and identified additional domains of SMP expression within the shell field. Given that the SMPs we examined were initially found in adult tissue, we hypothesize that the larval shell field regionalization of SMPs may correspond to similar regions in the adult mantle.

Different cell populations of the shell field have been identified in the bivalve *Crassostrea gigas* [79] and in multiple gastropod species [24, 26, 65, 80]. In the whelk *Tritia obsoleta*, a band of cells with high proliferation rate occupies the outermost portion of the shell field, which we refer to as the “shell field edge”, also known as the “leading edge” or “aperture growth zone” [26]. We examined the spatial-temporal expression of multiple SMPs within the same individual to identify distinctive SMP-expressing cell populations. We identified *CaSMP3* expressing cells located throughout the shell field, *CaSMP3* + and *CaSMP20* + cells in the inner edge, and *CaSMP20* expressing cells in the outer edge. Interestingly, we also found CaSMP3 + cells in the larval statocysts, small sensing organs that contain calcified stones known as statoliths. Sleight (2023) recently hypothesized that non-skeletal biomineralizing cells, such as those found in the statocyst, may represent an evolutionary precursor to biomineralizing cells in the molluscan shell field. The expression of *CaSMP3*, *CaSMP5*, *CaSMP12*, and *CaSMP4* in the statocysts of *C. atrasolea* larvae supports this hypothesis, and suggests that there are a subset of conserved SMPs shared between the statocyst and shell field. Our study provides a characterization of different SMP-expressing cell populations, and SMP regionalization within the shell field of *C. atrasolea*. To determine how cellular diversity contributes to the evolution of shell biomineralization, SMP-expressing cell populations should be compared in molluscs with diverse biomineralized structures.

### Evolutionary lineage of SMPs partly explains shell field modularity

One way by which shell diversification could have arisen is through the incorporation of novel SMPs, or co-option of conserved SMPs, in the biomineralization gene regulatory network. SMPs that have homologs outside of molluscs, for example in cnidarians or vertebrates, are considered ‘highly conserved’ genes likely inherited from a distant last common ancestor of metazoans, or bilaterians, respectively. Because the functions of these highly conserved genes have often been characterized in genetic model organisms, we know that their proteins mediate fundamental cellular processes like calcium regulation (e.g. calmodulin) or bicarbonate production (e.g. carbonic anhydrase) that may have been co-opted for biomineralization functions [2, 17, 30, 40, 41, 66, 81]. We found that the majority of *C. atrasolea* SMPs broadly-expressed in the shell field belonged to metazoan orthogroups (*n* = 6/7), and the majority of SMPs with a more restricted shell field edge expression were lineage-restricted to Mollusca or Gastropoda (*n* = 4/5). Although it is a small sample size, the trend may suggest that SMPs in Gastropod-specific and Mollusca-specific orthogroups tend to be expressed in more specific regions of the shell field. There are exceptions to this trend; for example, SMP20 is a metazoan SMP that was found restricted to the shell field edge, and SMP17 is a gastropod-restricted SMP, with a broader shell field expression domain. It is important to note that we have yet to fully characterize all species-restricted SMPs to understand how their evolutionary lineage affects shell regionalization. Our preliminary data show that the shell field regionalization of *C.fornicata*-specific *CfSMP6* (OG0088976) varied between different larval stages (supplementary fig. S21, Supplementary Material). The expression of *CfSMP6* was restricted to fewer shell field regions at the veliger stage compared to the late organogenesis stage of larval development (supplementary fig. S21, Supplementary Material). Although this represents only one species-restricted SMP, this may suggest dynamic regionalization of species-restricted SMPs during larval development. Based on our data, we hypothesize that highly conserved, metazoan SMPs involved in fundamental biomineralization processes [2], tend to be expressed in multiple shell field regions (i.e., broader shell field and shell field edge expression) compared to more recently-diverged, lineage-restricted SMPs, which may be restricted to singular shell field regions (e.g., shell field edge). On the other hand, conserved SMPs with specific shell field and/or restricted shell field edge expression may have adopted specialized roles in molluscan biomineralization by acquiring new expression domains. To test this hypothesis, future work can now compare the expression of conserved metazoan SMPs we have identified in *Crepidula* across multiple metazoan phyla.

Examining both the evolutionary age and regionalization of SMPs during larval shell development allows us to more precisely test hypotheses about the mechanisms that underpin mollusc shell divergence. Together, our findings point to the possibility that other mechanisms, such as changes to the modular expression of SMPs, also contribute to shell diversification. Future studies should prioritize broad taxon sampling, interspecies comparisons, and data accessibility.

## Conclusions

Here we present an evolutionary and developmental framework to allow future interspecies comparisons of molluscan biomineralization. We used orthogroup analysis to determine the evolutionary lineage of previously identified adult SMPs in *C. fornicata*, and found only 5% of those SMPs to be species-restricted. This small percentage suggests that there may be other drivers of the molluscan shell diversification beyond novel or species-specific SMPs. HCR analysis of a subset of SMPs in *C. atrasolea* larvae demonstrates expression in both the adult mantle and the larval shell field, suggesting that the adult and larval SMP complements may be more similar than those reported in other taxa. These findings highlight similarities in the SMP repertoires of adult and larval biomineralization. We also show that both highly conserved and lineage-restricted SMPs show distinct shell regionalization patterns; conserved SMPs tend to have broader expression throughout the shell field, while lineage-restricted SMPs show more restricted expression in the shell field edge. Together, these results suggest that the evolutionary age of an SMP may influence the modular shell field expression of SMPs during shell field development. Future gene perturbation experiments can use the *Crepidula* SMPs we have described to test the function of specific SMPs in biomineralization. Our work highlights the importance of integrating knowledge across taxa and developmental stages to better understand the role of SMPs in molluscan biomineralization and its diversification.

## Electronic supplementary material

Below is the link to the electronic supplementary material.

Supplementary Material 1

Supplementary Material 2

Supplementary Material 3

Supplementary Material 4

Supplementary Material 5

## Data Availability

The IsoSeq raw reads used to assemble the *C. atrasolea* transcriptome are currently available through NCBI’s SRA database under BioProject PRJNA1018959, and the assembled hybrid transcriptome was deposited to DDBJ/ENA/GenBank under the accession GKTO00000000. The version described in this paper is the first version, GKTO01000000. Orthofinder, Kinfin, and their accompanying input files and commands for job submission are available through Dryad (10.5061/dryad.zpc866tf1). The SMP sequences used to design HCR probes are available through NCBI's GenBank (accession numbers PP639282- PP639299).
