# Supplementary material for: The adult shell matrix protein repertoire of the marine snail Crepidula is dominated by conserved genes that are also expressed in larvae
Source: BMC Ecol Evol. 2024 Sep 14;24:120. doi: 10.1186/s12862-024-02237-y (PMC11401363; doi:10.1186/s12862-024-02237-y)
Supplement: Supplementary file 3 — Supplementary Material 3 [file 12862_2024_2237_MOESM3_ESM.docx]

**Supplementary Methods**

**S.1 Dissection of adult organs for RNA extraction**

For organ dissection, a single adult female was placed into a 100 x 20 mm petri dish (Corning #430167) and relaxed in 7.5% magnesium chloride (dissolved in filtered natural seawater; Sigma-Aldrich #M7304) at room temperature for 30 minutes prior to undergoing dissection. After relaxation, fine forceps (Dumont #6, Fine Science Tools) were used to gently depress the foot from the shell deck followed by gentle pulling on the foot to remove the remaining organs found within the mantle cavity of the shell. Surgical scissors were used to remove the head organ at the intersection between the neck and the anterior margin of the foot. The anterior dorsal mantle (extending to around one third the length of the mantle cavity aperture) and the mantle border surrounding the foot, were dissected using both forceps and surgical scissors. The foot and visceral mass were separated from one another at their intersection between shell deck and inner mantle cavity. All four organs (mantle, foot, head, and visceral mass) were each placed into 1.5 ml tubes for RNA extraction.

**S.2 Iso-Seq RNA Sequencing**

***S.2.1 RNA quality and quantity***

Quantity and quality of RNA was conducted using a NanoDrop spectrophotometer. In total, 1 µg of RNA was collected for each of the six samples (Samples 1-6). For embryonic samples (Samples 1-3), 0.5 µg of RNA was combined from each of their respective two pools of embryos. For Sample 4, 1 ug of RNA was taken from the adult male. For Sample 5, 1 µg of RNA was taken from the mantle tissue. Lastly, for Sample 6, 250 µg of RNA each was taken from the head, foot, gill, visceral mass, and mantle.

***S.2.2 Iso-Seq RNA preprocessing and clustering***

The initial processing and clustering of the IsoSeq data was performed by Roy J. Carver Biotechnology Center at the University of Illinois at Urbana-Champaign using SMRTLink V10.1.0. Briefly, Circular Consensus Sequencing (CCS) analysis was first done using the “ccs” command with parameters “--min-passes 3 --min-rq 0.999”. Sample demultiplexing was then performed using the “lima” command with parameters “--ccs --same --split-bam-named”. This resulted in six bam files, each storing the reads from one of the six samples sequenced. Next, SMRTLink linux-toolkit V10.0.0 was used to 1. remove primers (lima --isoseq … primers.fasta …), 2. classify strandness and trim polyA (isoseq3 refine --require-polya), and 3. de novo cluster the transcripts (isoseq3 cluster --use-qvs) for each sample. The final output is a fasta file containing full length consensus isoforms for each sample. We additionally merged the bam files for all samples and repeated the clustering step using “isoseq3 cluster” command to generate a consensus isoform fasta file across all samples. This fasta file was used in the subsequent hybrid transcriptome construction.

**S.3. Hybrid transcriptome assembly and annotation**

***S.3.1 Obtaining short read transcriptome data***

Briefly, the short read transcriptome contains Illumina HiSeq 2500 sequencing results of *Crepidula atrasolea* cDNA library from approximately ~100-200 embryos from each of the following stages: 1) 2- cell to 25-cell, early cleavage-stage embryos, 2) later cleavage-stage embryos including the formation the 4a-4c micromeres, 3) a mix of more advanced cleavage stages undergoing compaction and initiating gastrulation, 4) early gastrula stage embryos, 5) flattened mid to late gastrula stages undergoing epiboly, 6) embryos undergoing elongation, 7) embryos initiating organogenesis, and 8) more advanced embryos with curved shells.

***S.3.2 Hybrid transcriptome assembly with both short and long read libraries***

Since the short read library was prepared with Illumina's TruSeq Stranded mRNAseq Sample Prep kit (Illumina), an additional --ss rf option was specified in the command to specify the strandness of the short read paired-end libraries (first read in pair corresponds to reverse gene strand). In summary, the main command for assembling the hybrid transcriptome is as follows: rnaspades.py -1 ${short_R1} -2 ${short_R2} --ss rf --pacbio ${long_ccs} -o ${result_dir}, where “short_R1” and “short_R2” are paths to the read1 and read2 fastq files from the short read library, “long_ccs” is the path to the Iso-seq CCS reads in fastq format, and “result_dir” specifies the output directory. All commands were executed on the Expanse cluster at the San Diego Supercomputer Center (SDSC). rnaSPAdes returns three transcriptomes with soft- default- and hard- filters, each more stringent in keeping high quality, long length transcripts. We used the default filtered results for subsequent steps as recommended in the rnaSPAdes manual.

***S.3.3 Combining highly similar transcripts***

There are highly similar transcripts in the assembled transcriptome that likely represent the same isoform from the same gene, but with small differences due to polymorphism among the non-inbred population used as sample, or technical artifacts during PCR and sequencing. To remove highly redundant transcripts, we ran CD-HIT (version 4.8.1) (Fu et al. 2012) to cluster transcripts in the transcriptome with similarity >95%; only one transcript from each cluster is then kept in the transcriptome. This analysis was done on the SDSC Expanse cluster. The option “-r 0” was used with the cd-hit-est command to perform only +/+ strand alignment during clustering. CD-HIT clustering reduced the total number of transcripts in the transcriptome from 848,512 to 699,466, while maintaining a high BUSCO completeness (99.2% against Metazoan database and 91.7% against Mollusca).

***S.3.4 Removing foreign transcripts***

We used alien index code to remove any foreign transcripts from our sequencing data (Ryan 2014). This was aimed to remove any potential contamination introduced in the RNA extraction and RNA sequencing steps. We used the supplied metazoan protein sequence database (<http://ryanlab.whitney.ufl.edu/downloads/alien_index/>) as the “non-alien” sequence database. We also used the supplied non-metazoan protein sequence database with the addition of *Diacronema lutheri* as the “alien” sequence database (Nelson et al. 2021). Each transcript is given an alien index score based on the difference between the E-values of the blast to the metazoan and non-metazoan databases. We removed transcripts with an alien index of 45 or higher, meaning they were likely to be contaminant non-metazoan sequences.

***S.3.5 Inferring coding sequences***

We inferred the coding sequences (CDSs) in the assembled and cleaned transcriptome using TransDecoder (version 5.5.0) (Haas and Papanicolaou 2015) on SDSC Expanse cluster. “-m 50” option was used with the “TransDecoder.LongOrfs” command to retain only the open reading frames (Orfs) whose protein products are at least 50 amino acids long. The “TransDecoder.Predict” command was then run to predict likely CDSs and their protein/peptide products. CDSs and their products were then filtered to keep only the ones on the sense strand. This resulted in 241,276 sense CDSs and their corresponding proteins/peptides from 195,575 transcripts in the transcriptome. We performed BUSCO analysis on these 195,575 transcripts hosting predicted sense CDSs, and confirmed the persisting high completeness (99.2% against Metazoa and 91.5% against Mollusca databases), while the remaining transcripts in the transcriptome (without long, sense CDS) scored low completeness (6.5% Metazoa and 3.3% Mollusca). The collection of the resulting protein/peptide sequences are used as the proteome for the subsequent analyses.

***S.3.6 Protein annotations***

We used two strategies to annotate proteins and peptides in the proteome constructed per the previous section. First, we scanned the protein/peptide sequences for protein families and domains documented in the InterPro database (Paysan-Lafosse et al. 2023). This was accomplished by running the InterproScan software (version 5.52) (Jones et al. 2014) on the Bridges-2 supercomputing platform at the Pittsburgh Supercomputing Center (PSC). The list of annotation sources used in InterproScan was specified in the command with the -appl option, which include CDD (v3.18), Coils (v2.2.1), Gene3D (4.3.0), Hamap (v2020_05), MobiDBLite (v2.0), PANTHER (v15.0), Pfam (v33.1), PIRSF (v3.10), PIRSR (v2021_02), PRINTS (v42.0), SFLD (v4), SMART (v7.1), SUPERFAMILY (v1.75), and TIGRFAM (v15.0). This approach annotated 126,224 out of the 241,276 entries in the proteome.

As a complementary approach, we blasted each protein/peptide in the *C. atrasolea* proteome against the NCBI Invertebrate Reference Sequence database (RefSeq) (Pruitt et al. 2012). First, a local BLAST database was made for the Invert-RefSeq database on the Bridges-2 cluster (RefSeq database downloaded: May 13, 2022). Next, the *C. atrasolea* proteome was split into three files: two files each containing 100,000 sequences, and the third file containing 41,276 sequences. Pairwise similarity searches were performed for the proteome against the RefSeq invertebrate database using the “BLASTP” command with the following parameters: “-max_target_seqs 10 -outfmt 6 -evalue 1e-5”. Three tabulated output files were generated which were concatenated into a single file. This approach annotated 86,287 of 241,276 entries in the proteome.

**S.4 Details on species proteomes and data curation for orthology inference**

Sequence accessions for each transcriptome and biomineral proteome were renamed sequentially and include the first letter of their genus name followed by the first few letters of their species names (e.g. Catra_1, Catra_2, Catra_3). Lookup tables were made to match the renamed accession to their original transcriptome accession.

**S.5 Hybridization chain reaction in *Crepidula atrasoslea***

Embryos were aliquoted into 1.5 ml tubes with their respective probe set and amplifier. Fixed embryos were stored in methanol and were re-hydrated into 5X SSCT buffer following 5 minute 75% methanol 25% 5X SSCT, 50% methanol 50% 5X SSCT, 25% methanol 75% 5X SSCT, and two 100% 5X SSCT washes. Samples were pre-hybridized in 100 μl of probe hyb buffer (30% formamide; # JT4028-1, 5X sodium chloride sodium citrate, 9 mM citric acid (pH 6.0)**:** # C1909, 0.1% Tween 20, 50 μg/ml heparin: Sigma-Aldrich # H3393, 1X Denhardt’s solution: Thermo Scientific # AAJ63135AE, 10% dextran sulfate: Sigma-Aldrich # S4030) at 37°C for 30 minutes. After removing the probe hyb buffer, samples were incubated in 100 ul of the probe solution (1 pmol probe in probe hyb buffer) for 20-24 hours at 37°C. The probe solution was then removed from each sample and washed out with four 5 minute and two 30 minute rinses with probe wash buffer (30% formamide, 5X sodium chloride sodium citrate, 9 mM citric acid (pH 6.0), 0.1% Tween 20, and 50 μg/ml heparin) at 37°C. After washing out the probe, samples were rinsed twice with 5X SSCT for 5 minutes at 20°C. Between these washes, DNA amplifier hairpin sets (H1 and H2: Molecular Instruments<https://www.molecularinstruments.com/>) corresponding to the amplifiers used for each tube were snapped separately at 95°C for 90 seconds and then cooled for 30-32 minutes at room temperature before used for the hairpin solution (6 pmol of hairpin solution for each hairpin in 100 ul of amplification buffer per tube). After removing the 5X SSCT solution from each tube, the samples were incubated in the hairpin solution for 22-24 hours at 20°C. Hairpins were then removed by three consecutive 5 minute 5X SSCT washes.
