# Supplementary material for: The adult shell matrix protein repertoire of the marine snail Crepidula is dominated by conserved genes that are also expressed in larvae
Source: BMC Ecol Evol. 2024 Sep 14;24:120. doi: 10.1186/s12862-024-02237-y (PMC11401363; doi:10.1186/s12862-024-02237-y)
Supplement: Supplementary file 5 — Supplementary Material 5 [file 12862_2024_2237_MOESM5_ESM.docx]

**Figure S1.**
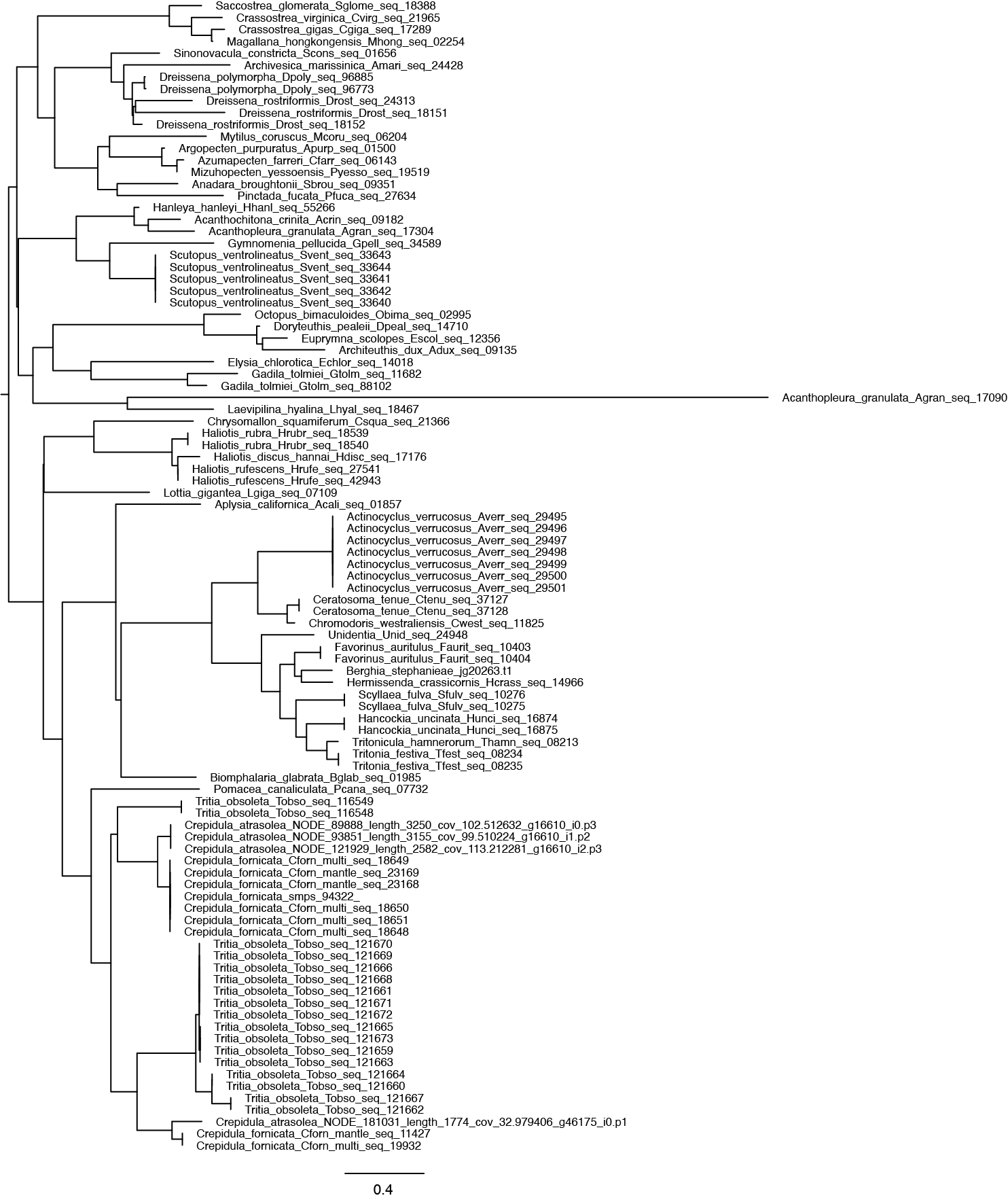


**Figure S1. Phylogenetic tree inference for *Ca94322* orthogroup OG0008650**. Orthogroup genes were aligned using MAFTT (version 7.221; Katoh et al. 2002) and FastTree (Price et al. 2009) was used tree inference. Scale bars indicate number of substitutions per site.

**Figure S2.**


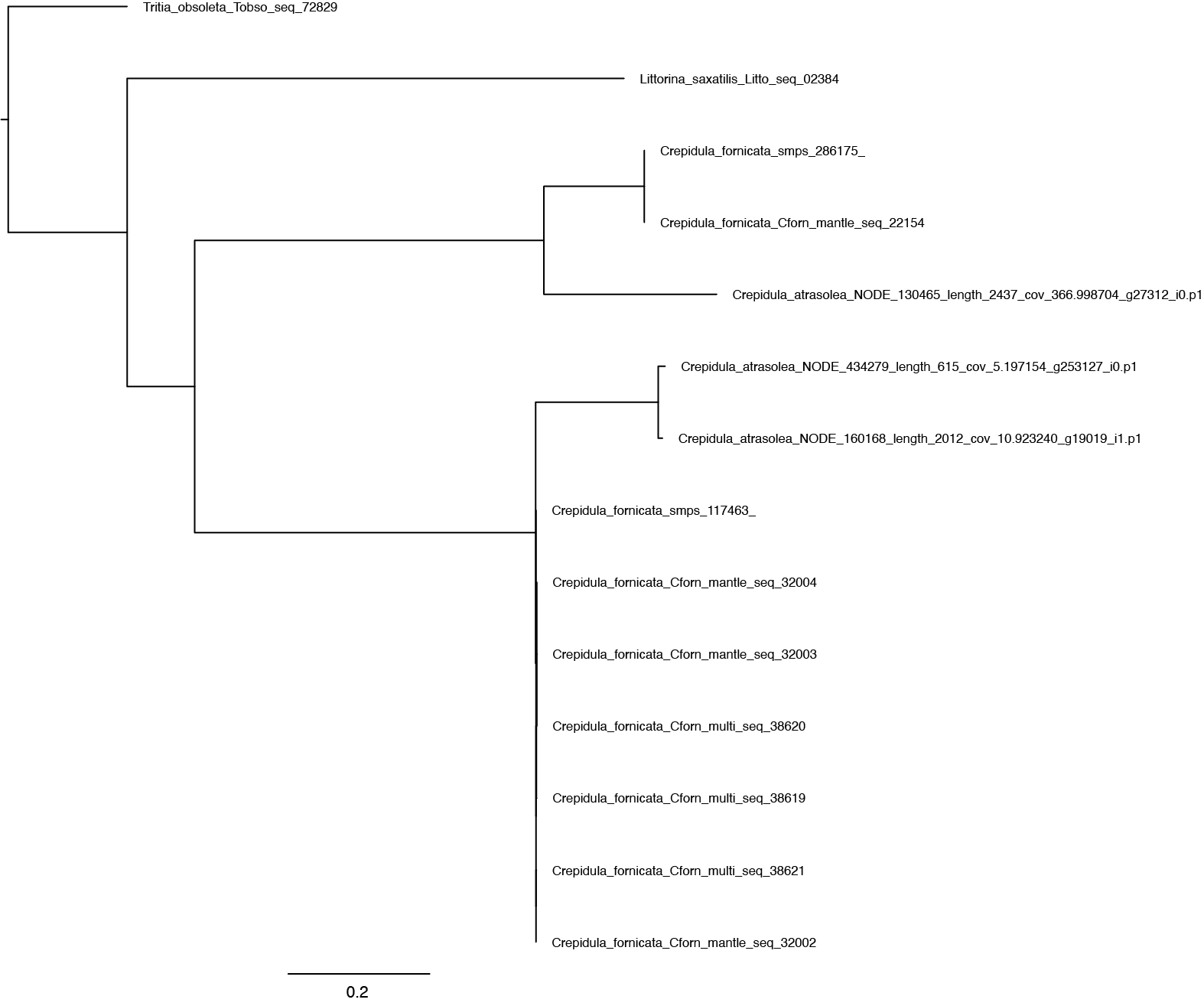


**Figure S2. Phylogenetic tree inference for *Ca117463* and *Ca286175* orthogroup OG0025994.** Orthogroup genes were aligned using MAFTT (version 7.221; Katoh et al. 2002) and FastTree (Price et al. 2009) was used tree inference. Scale bars indicate number of substitutions per site.

**Figure S3.**


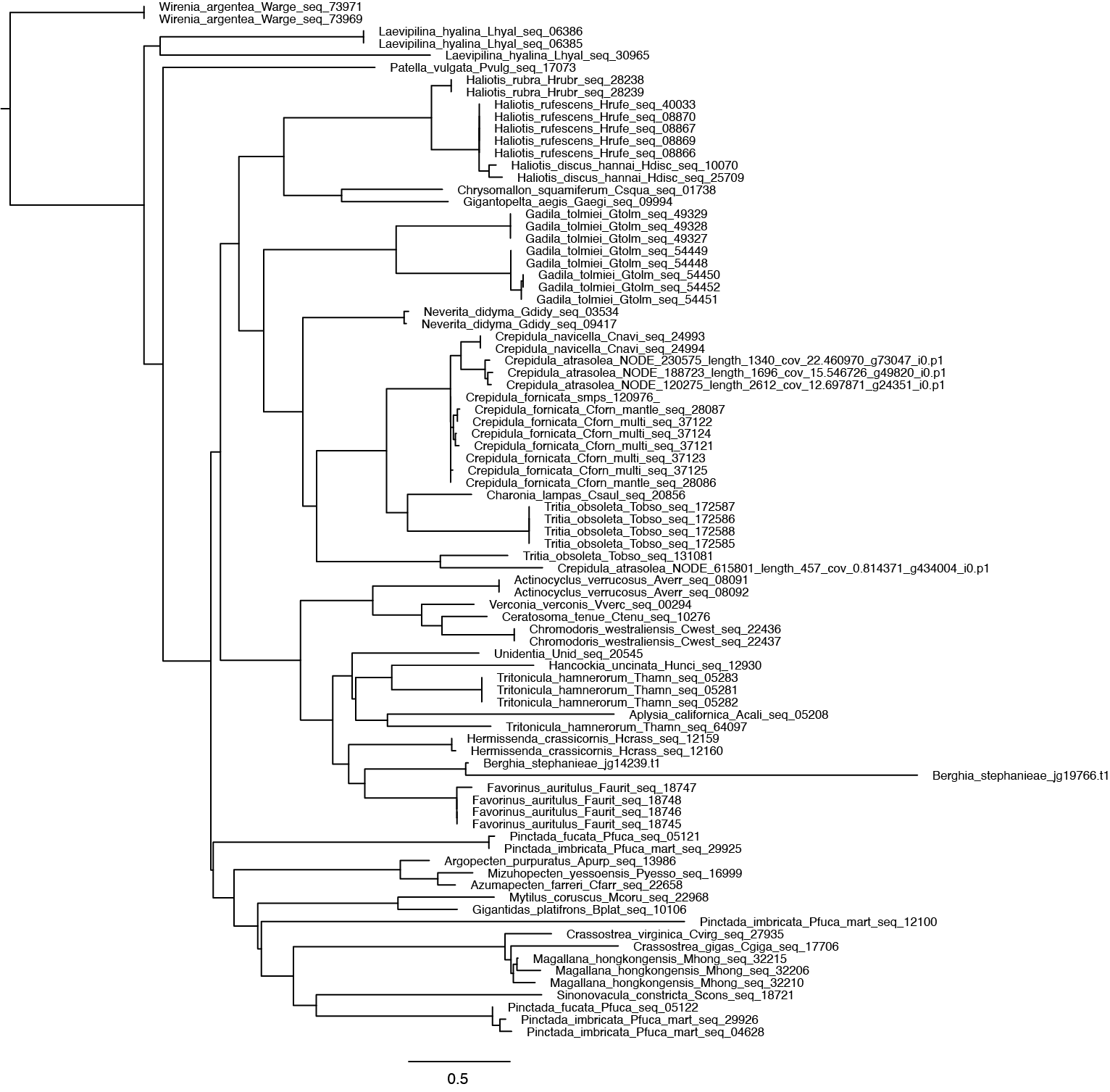


**Figure S3. Phylogenetic tree inference for *Ca120976* orthogroup OG0009176.** Orthogroup genes were aligned using MAFTT (version 7.221; Katoh et al. 2002) and FastTree (Price et al. 2009) was used tree inference. Scale bars indicate number of substitutions per site.

**Figure S4.**


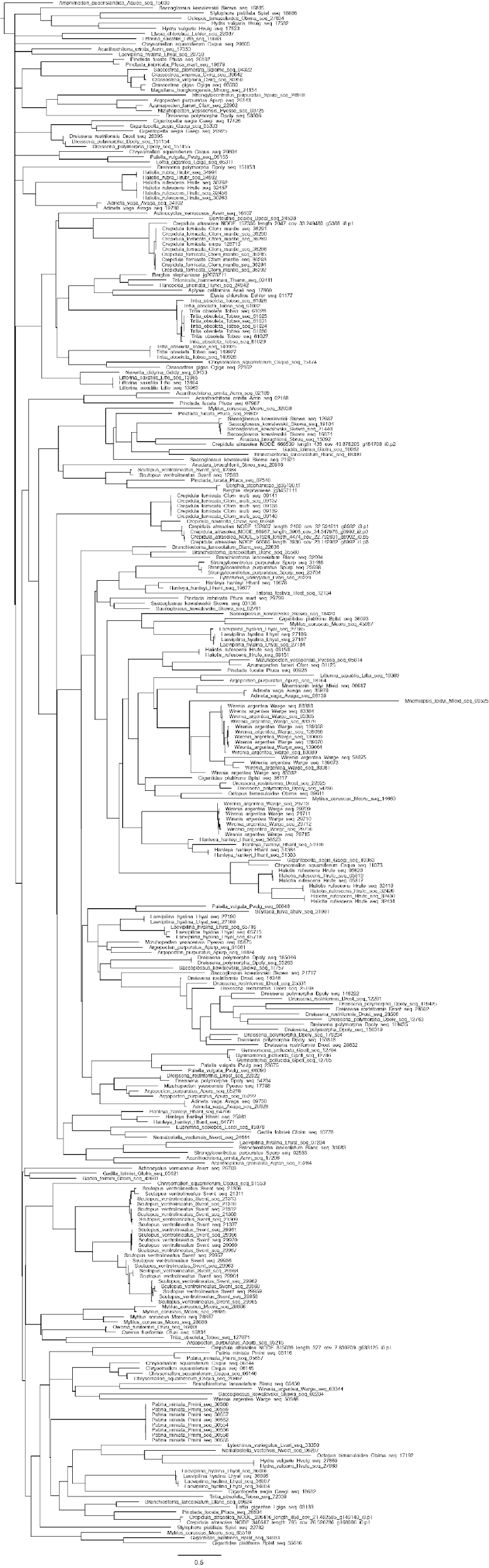


**Figure S4. Phylogenetic tree inference for *Ca128713* orthogroup OG0001737.** Orthogroup genes were aligned using MAFTT (version 7.221; Katoh et al. 2002) and FastTree (Price et al. 2009) was used tree inference. Scale bars indicate number of substitutions per site.

**Figure S5.**


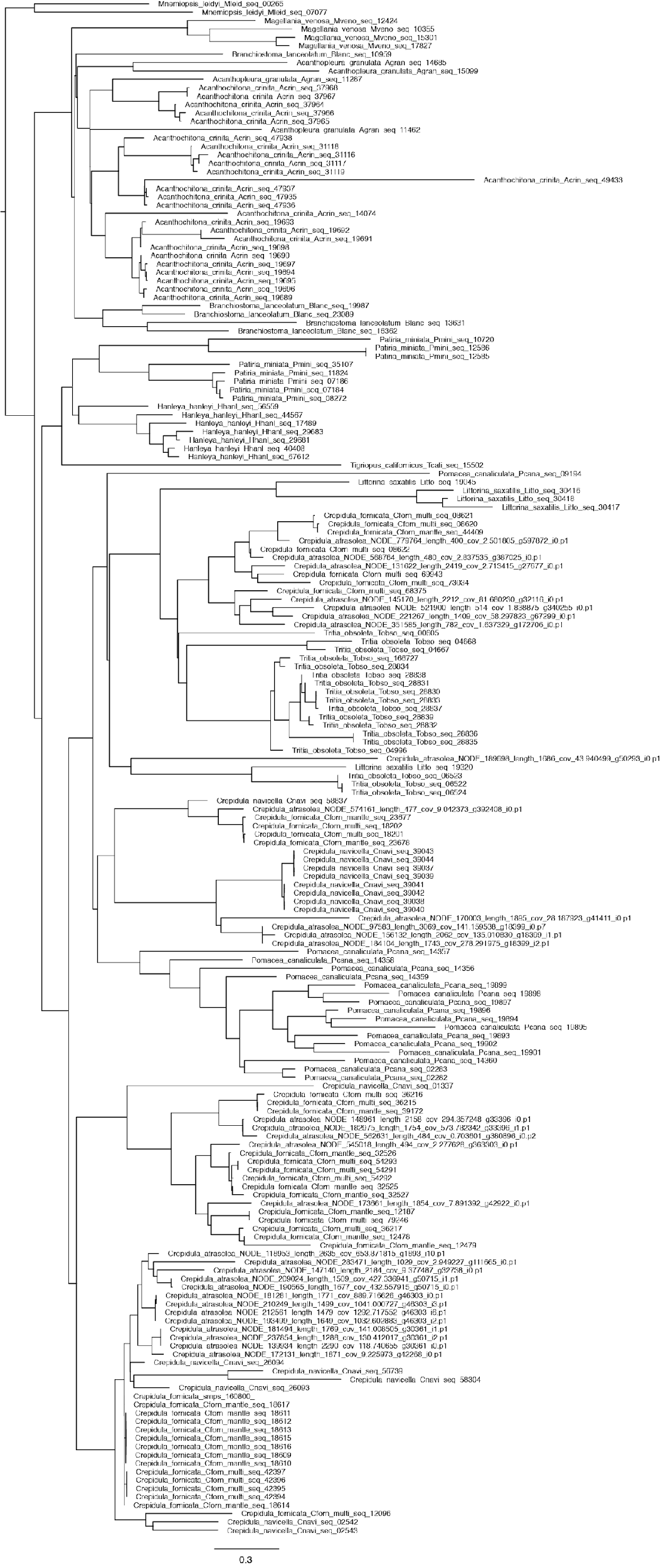


**Figure S5. Phylogenetic tree inference for *Ca160800* orthogroup OG0004218.** Orthogroup genes were aligned using MAFTT (version 7.221; Katoh et al. 2002) and FastTree (Price et al. 2009) was used tree inference. Scale bars indicate number of substitutions per site.

**Figure S6.**


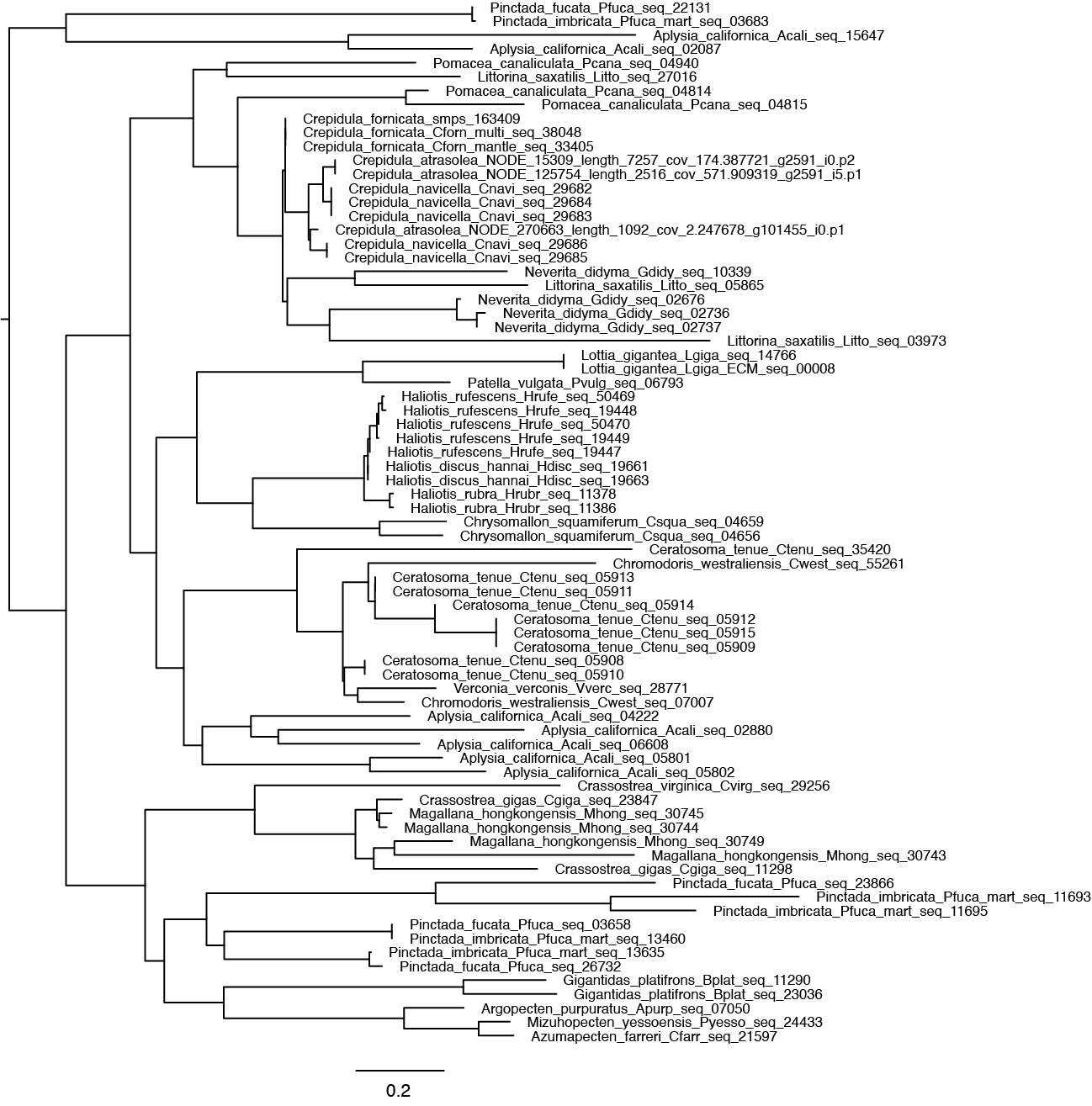


**Figure S6. Phylogenetic tree inference for *Ca163409* orthogroup OG0009593.** Orthogroup genes were aligned using MAFTT (version 7.221; Katoh et al. 2002) and FastTree (Price et al. 2009) was used tree inference. Scale bars indicate number of substitutions per site.

**Figure S7.**

**
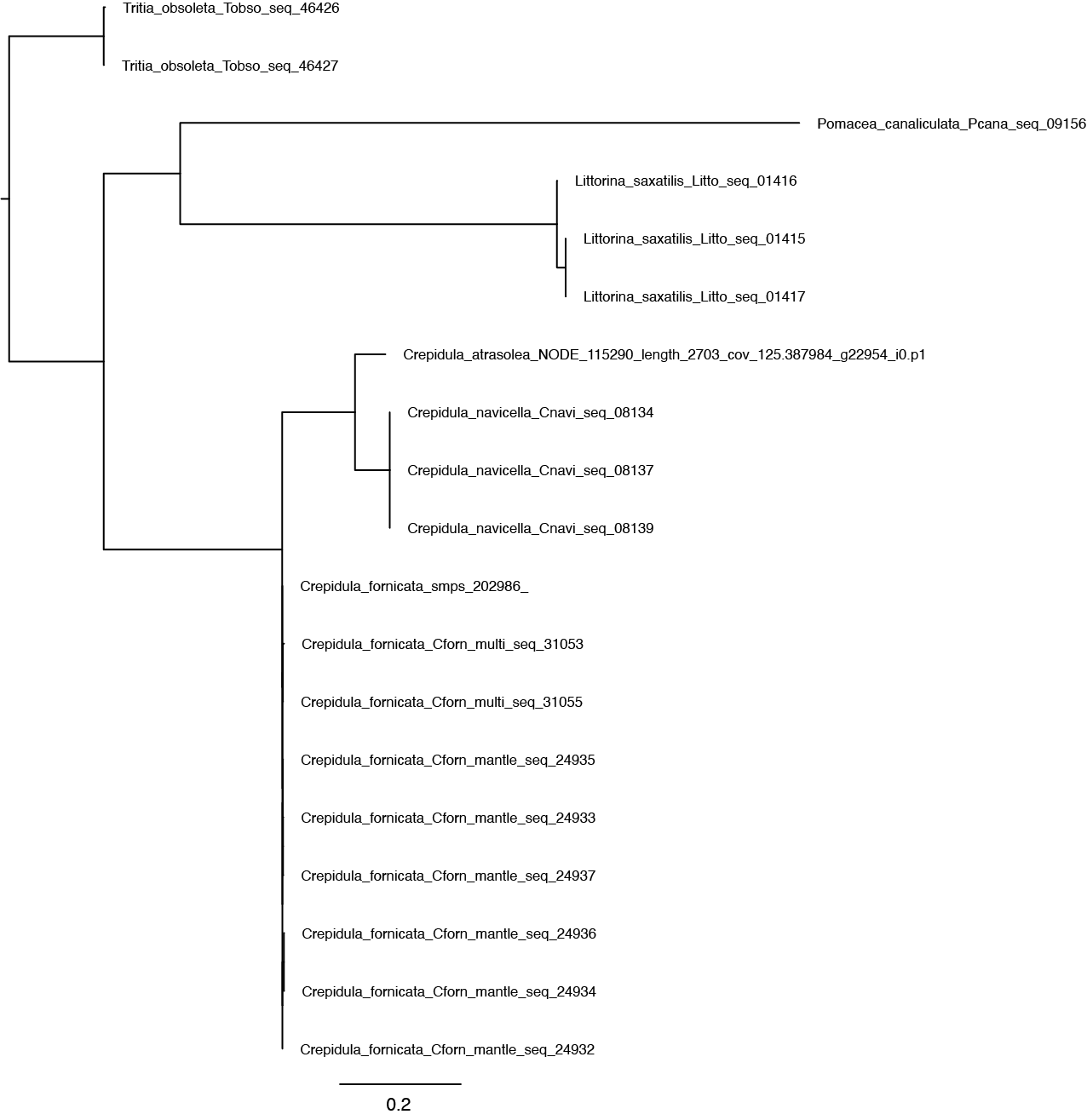
**

**Figure S7. Phylogenetic tree inference for *Ca202986* orthogroup OG0019902.** Orthogroup genes were aligned using MAFTT (version 7.221; Katoh et al. 2002) and FastTree (Price et al. 2009) was used tree inference. Scale bars indicate number of substitutions per site.

**Figure S8.**

**
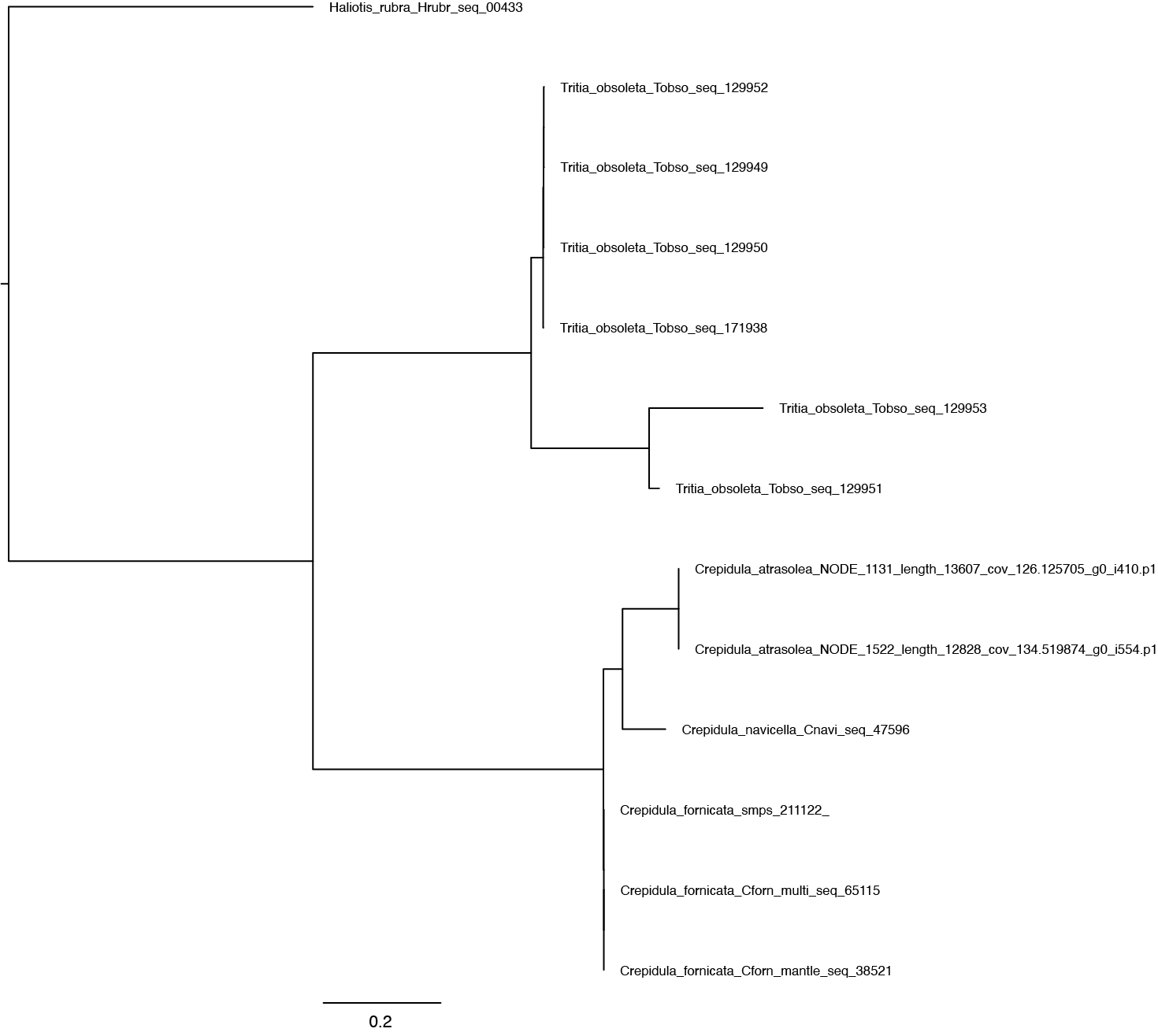
**

**Figure S8. Phylogenetic tree inference for *Ca211122* orthogroup OG0027870.** Orthogroup genes were aligned using MAFTT (version 7.221; Katoh et al. 2002) and FastTree (Price et al. 2009) was used tree inference. Scale bars indicate number of substitutions per site.

**Figure S9.**

**
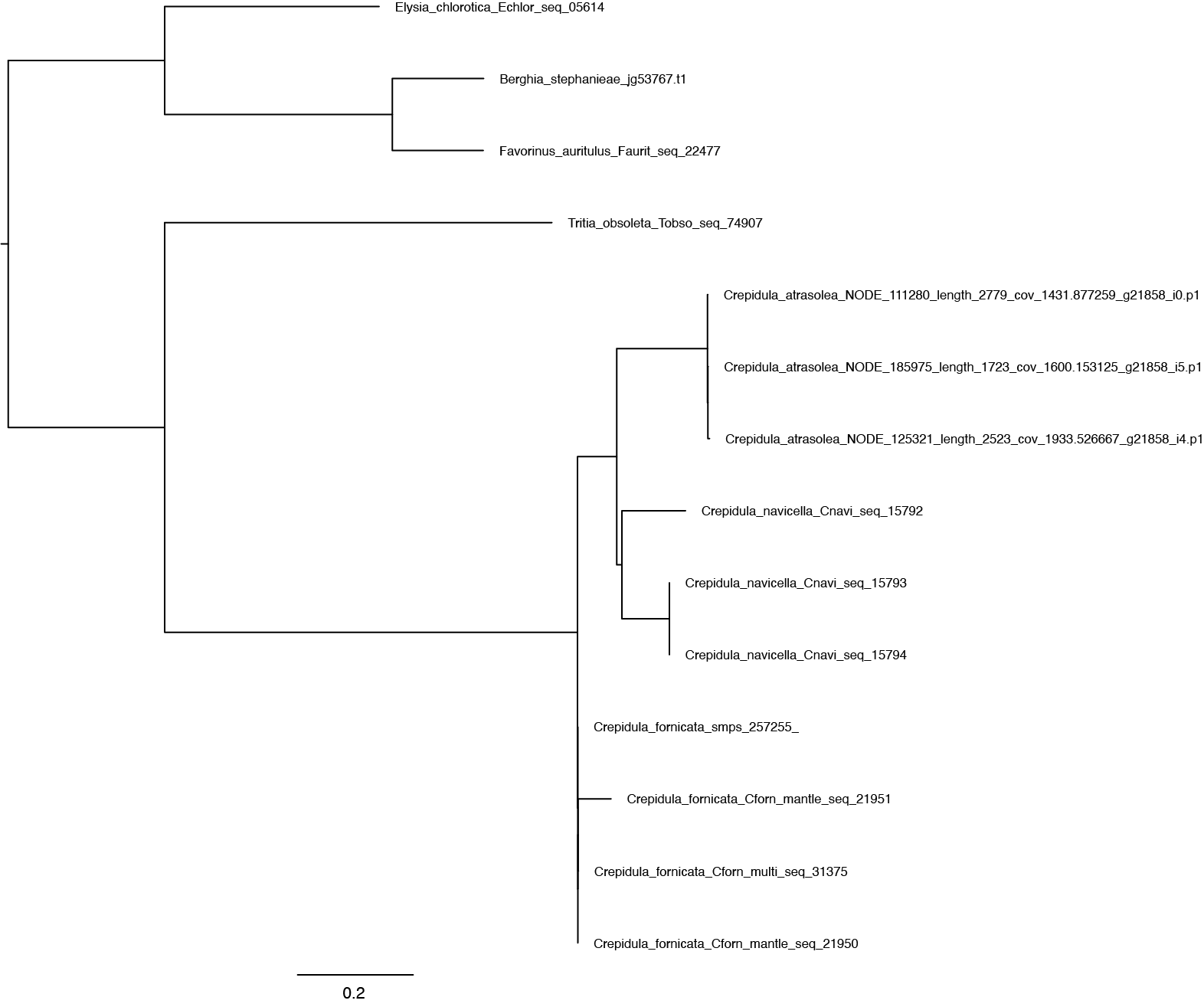
**

**Figure S9. Phylogenetic tree inference for *CaSMP1* orthogroup OG0025548.** Orthogroup genes were aligned using MAFTT (version 7.221; Katoh et al. 2002) and FastTree (Price et al. 2009) was used tree inference. Scale bars indicate number of substitutions per site.

**Figure S10.**

**
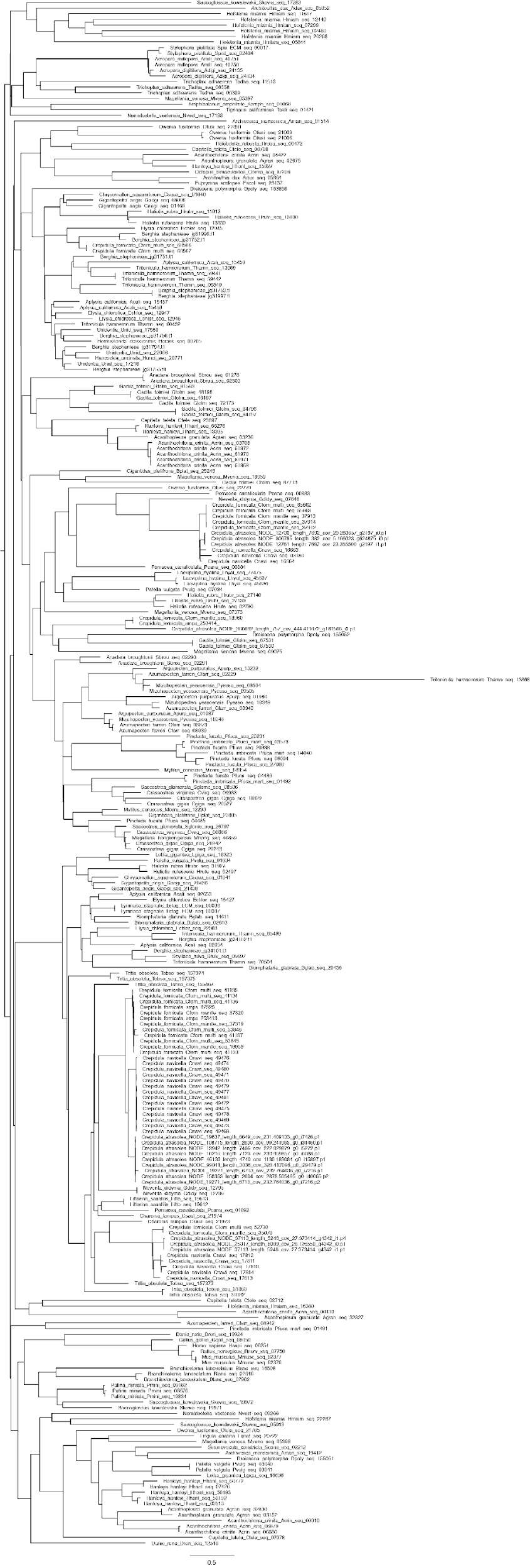
**

**Figure S10. Phylogenetic tree inference for *CaSMP3*, *CaSMP4*, *CaSMP5*, *CaSMP12* orthogroup OG0002026.** Orthogroup genes were aligned using MAFTT (version 7.221; Katoh et al. 2002) and FastTree (Price et al. 2009) was used tree inference. Scale bars indicate number of substitutions per site..

**Figure S11.**

**
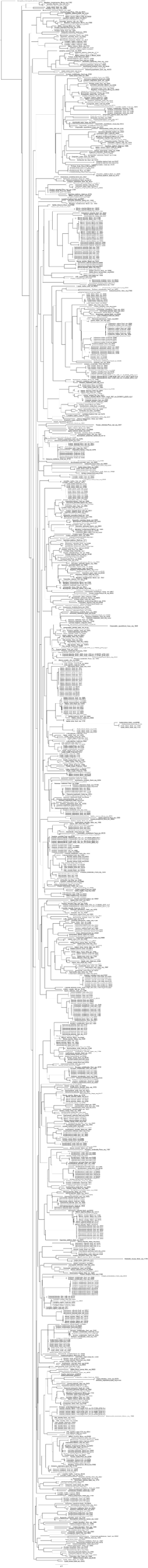
**

**Figure S11. Phylogenetic tree inference for *CaSMP9*, and *CaSMP10* orthogroup OG0000273.** Orthogroup genes were aligned using MAFTT (version 7.221; Katoh et al. 2002) and FastTree (Price et al. 2009) was used tree inference. Scale bars indicate number of substitutions per site.

**Figure S12.**

**
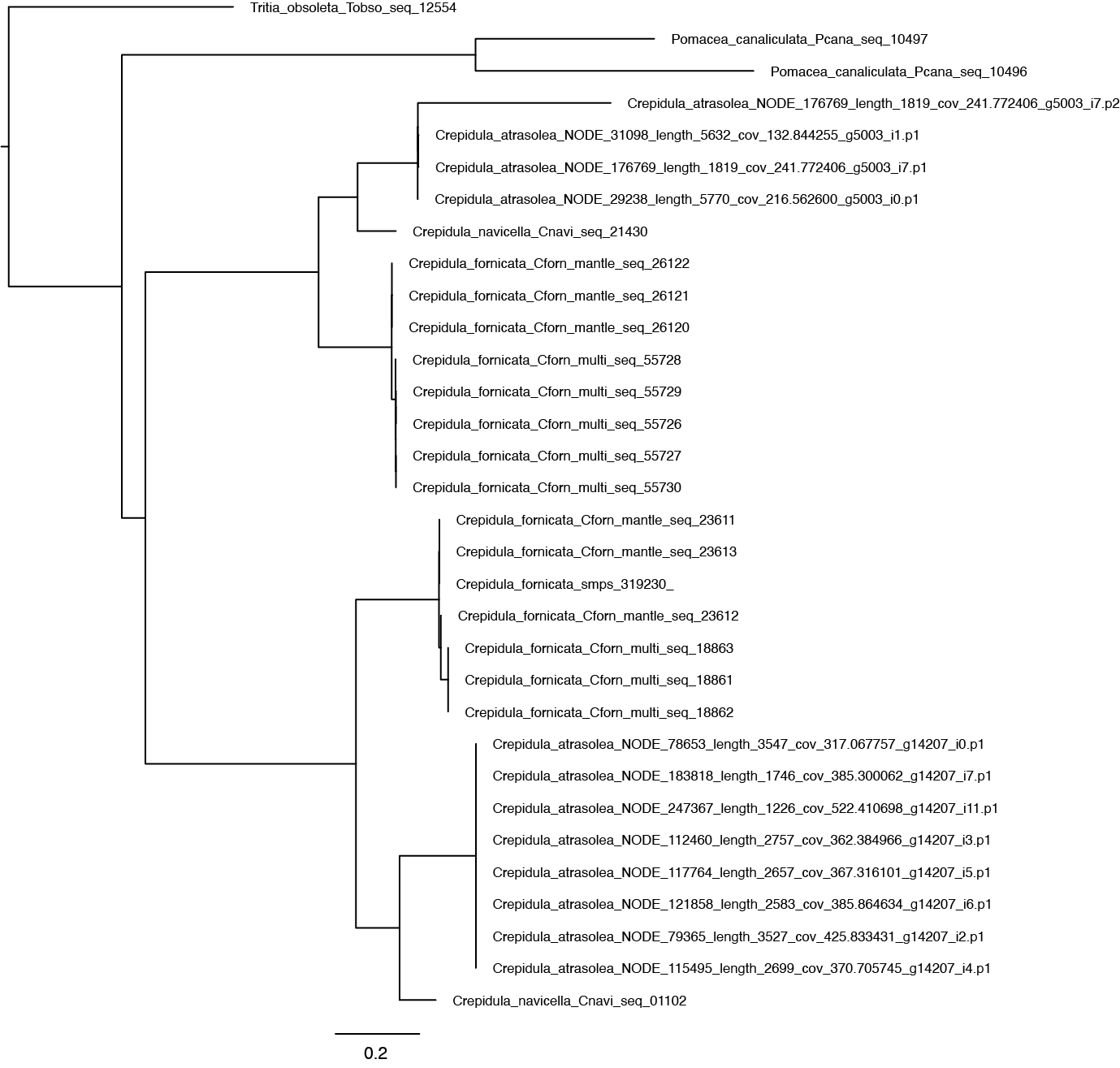
**

**Figure S12. Phylogenetic tree inference for *CaSMP17* orthogroup OG0013865 Tree**. Orthogroup genes were aligned using MAFTT (version 7.221; Katoh et al. 2002) and FastTree (Price et al. 2009) was used tree inference. Scale bars indicate number of substitutions per site.

**Figure S13.**

**
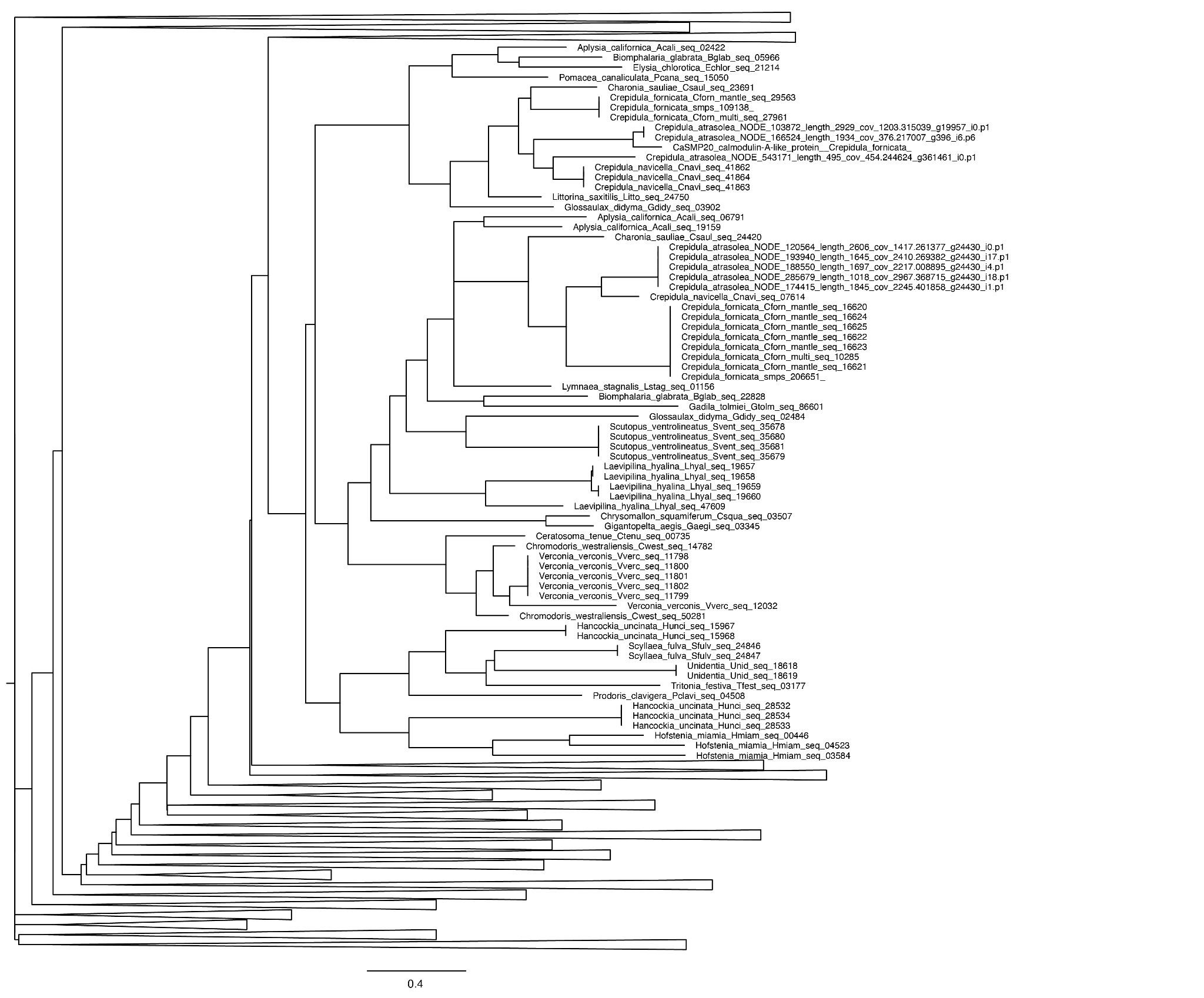
**

**Figure S13. Phylogenetic tree inference for *CaSMP20* orthogroup OG0000038.** Orthogroup genes were aligned using MAFTT (version 7.221; Katoh et al. 2002) and FastTree (Price et al. 2009) was used tree inference. Scale bars indicate number of substitutions per site.

**Figure S14**


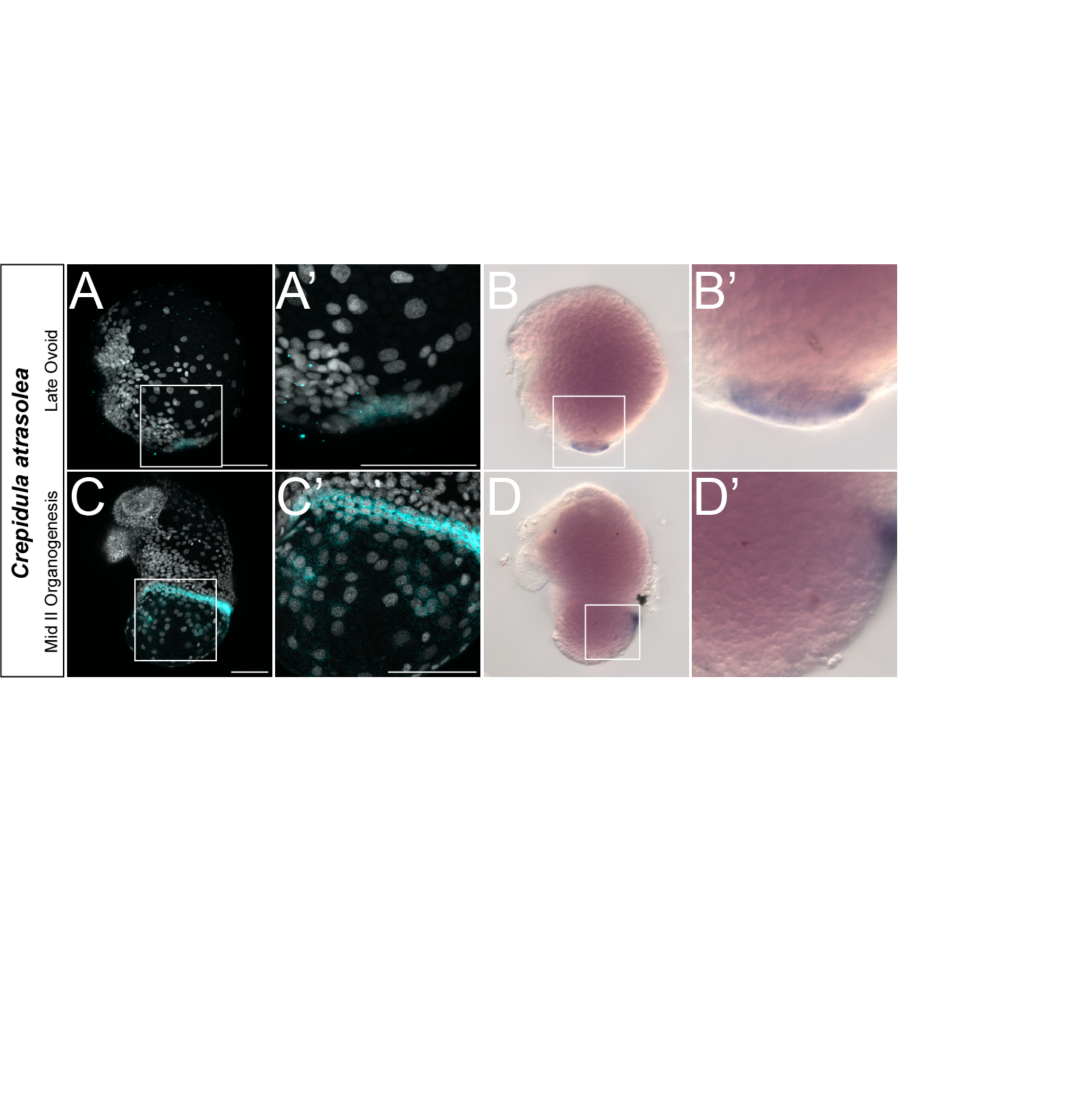


**Figure S14. Improved Detection of *CaSMP1* Expression with *In-situ* Hybridization Chain Reaction vs. Colorimetric *In-Situ*** **Hybridization**. Using *in-situ* hybridization chain reaction*, CaSMP1* mRNA expression was detected in the mantle edge and shell field of *C. atrasolea* embryos at the late ovoid (A) and mid II organogenesis (C) stages. Meanwhile, colorimetric *in-situ* hybridization detected expression only in the mantle edge at both late ovoid

**Figure S15.**


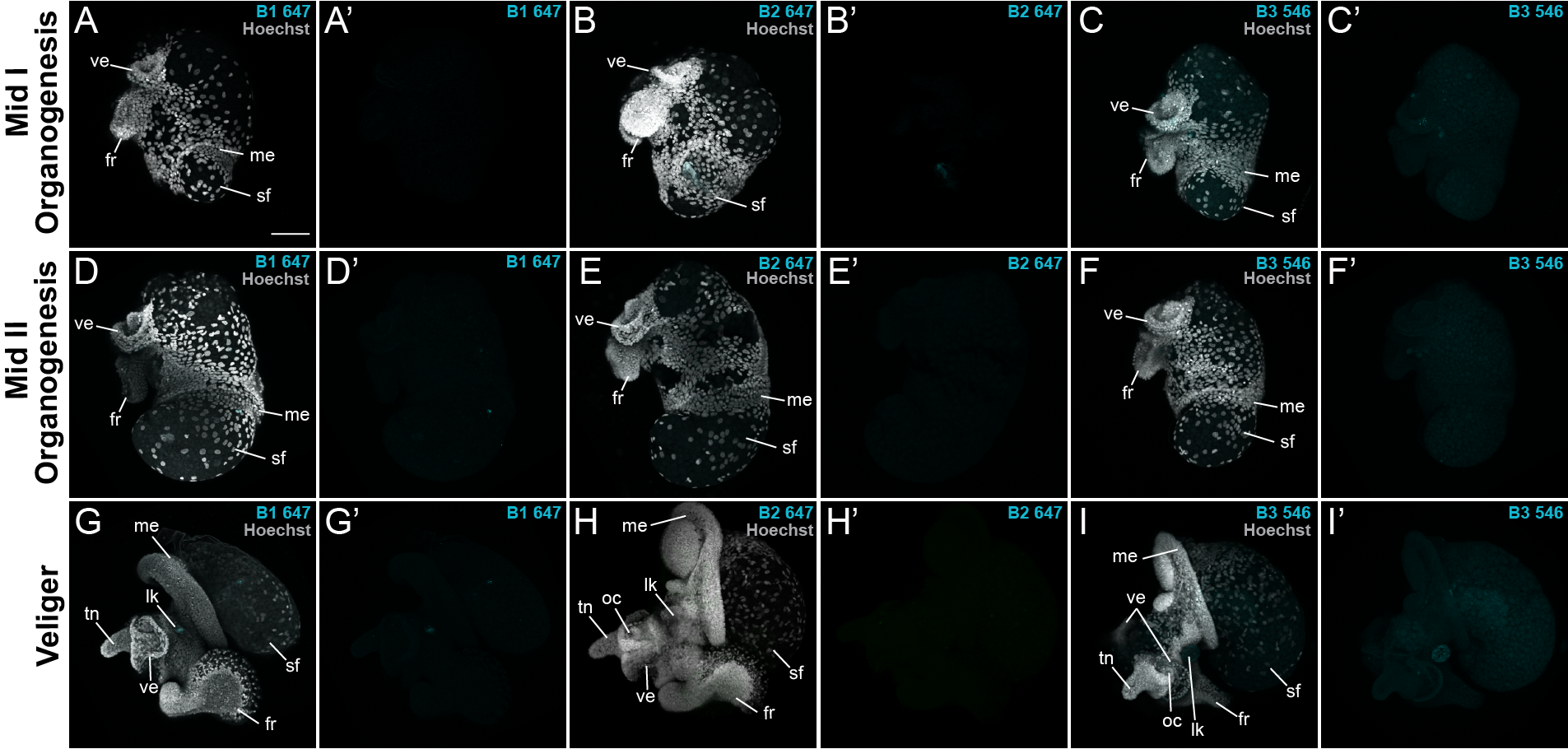


**Figure S15. *C. atrasolea* Hybridization Chain Reaction Controls**. Control *C. atrasolea* samples hybridized without a probe set at mid I organogenesis (A, B, and C), mid II organogenesis (D, E, and F) and veliger stages (G, H, and I). Control samples were incubated with either B1 647 (A, D, and G), B2 647 (B, E, and H), or B3 546 (C, F, and I) fluorophore hairpins. Hoechst is shown in gray and the fluorophore background signal in cyan. ft, foot; sf, shell field; tn, tentacles; ve, velar lobes; lk, larval kidney. Scale bar represents 100 μm.

**Figure S16**


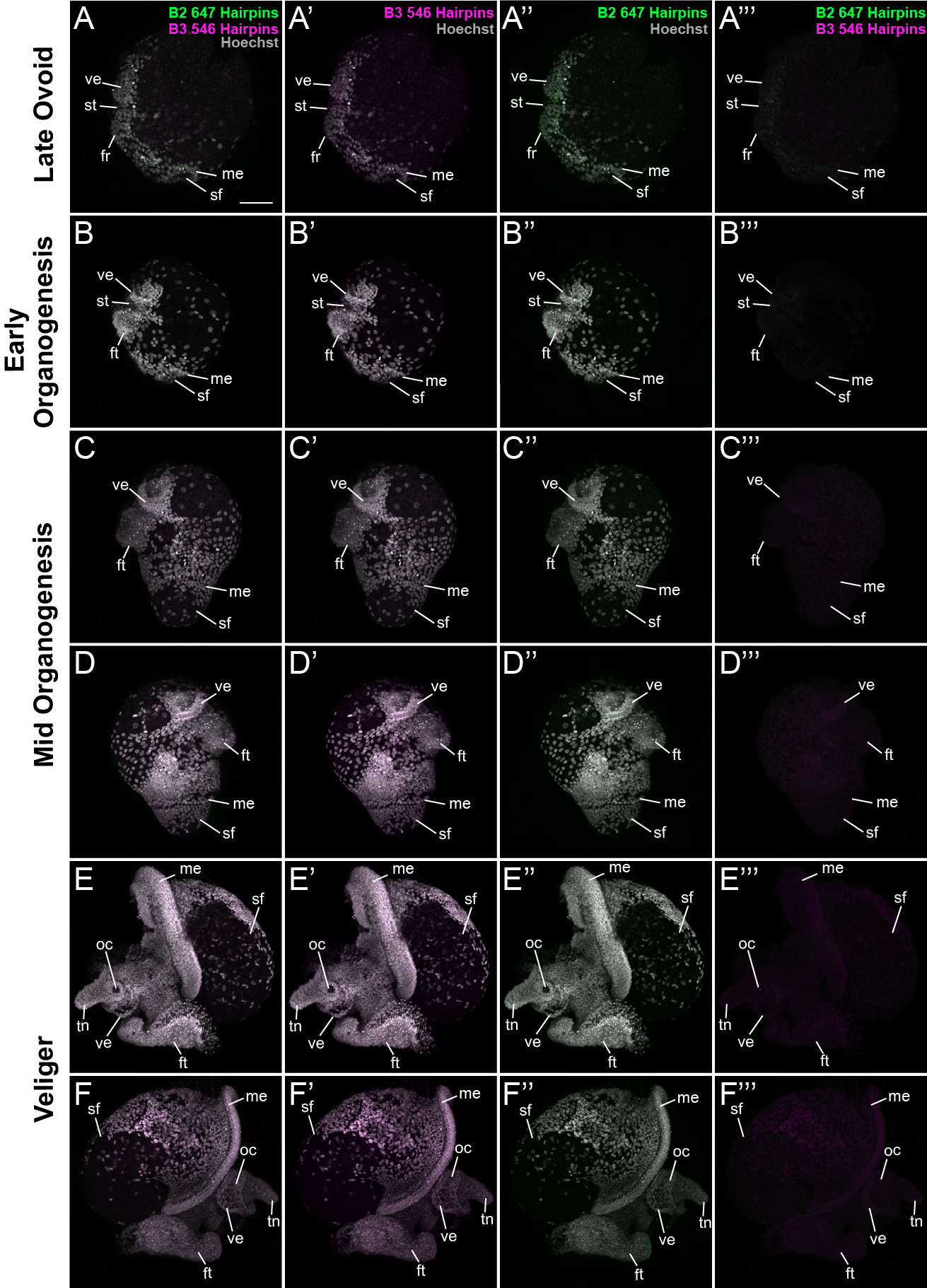


**Figure S16. *C. atrasolea* Hybridization Chain Reaction Multiplexing Controls**. Control *C. atrasolea* multiplexing samples hybridized without a probe set at late ovoid (A), early organogenesis (B), mid I organogenesis (C and D), and veliger stages (E and F). Control samples were incubated with both B1 647 (shown in green), and B3 546 (shown in magenta) fluorophore hairpins. Hoechst is shown in gray. ft, foot; sf, shell field; tn, tentacles; ve, velar lobes; lk, larval kidney. Scale bar represents 100 μm.

**Figure S17**


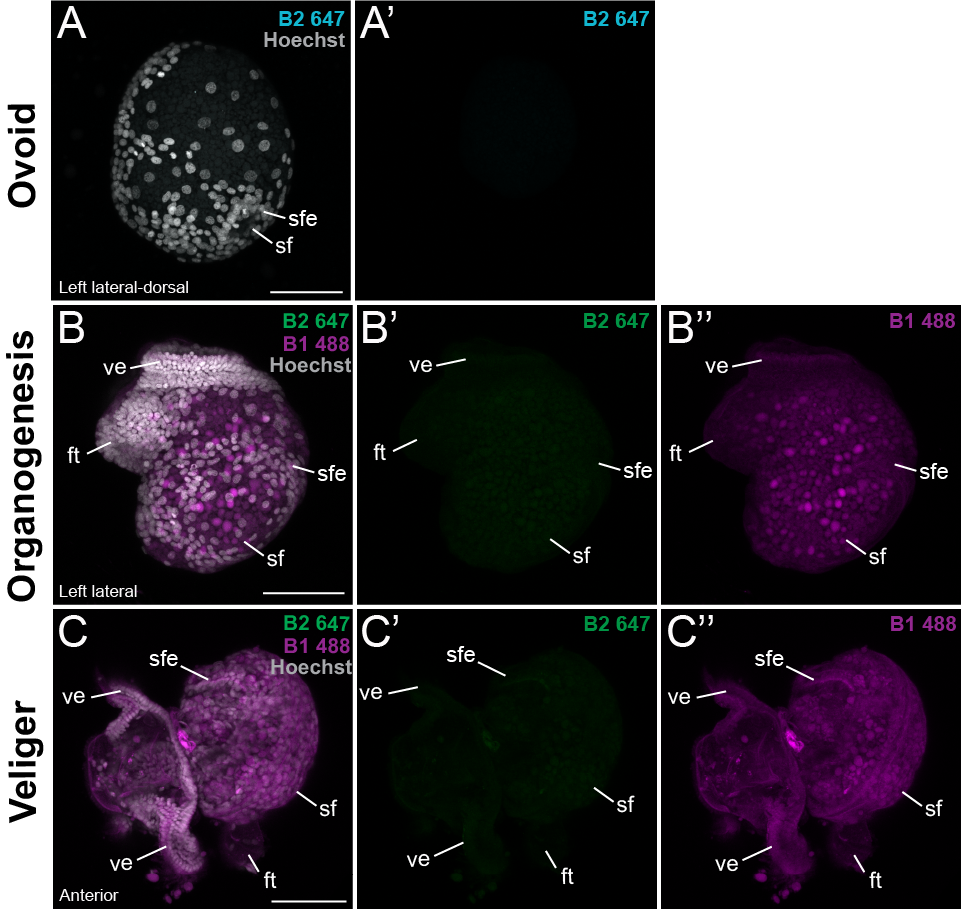


**Figure S17. *C. fornicata* Hybridization Chain Reaction Controls**. Control *C. fornicata* samples hybridized without a probe set at ovoid (A), organogenesis (B), and veliger (C) stages. Control samples were incubated with B2 647 only (A; shown in cyan) or both B2 647 (shown in green) and B1 488 (shown in green) fluorophore hairpins. Hoechst is shown in gray. ft, foot; sf, shell field; sfe, shell field edge; tn, tentacles; ve, velar lobes; lk, larval kidney. Scale bar represents 100 μm.

**Figure S18**


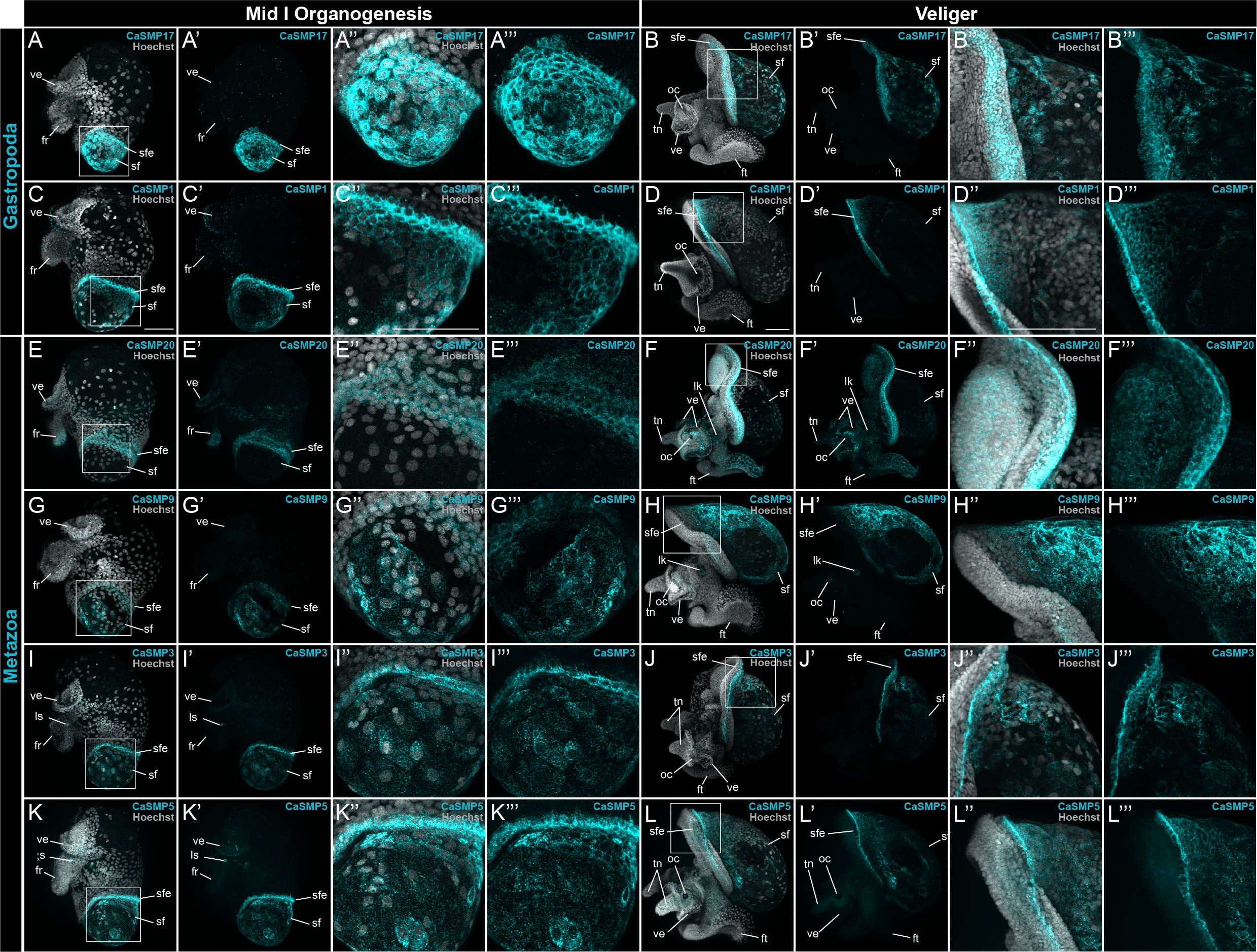


**Figure S18. Shell field expression of SMPs in *C. atrasolea* at organogenesis and veliger stages.** Using hybridization chain reaction, mRNA expression was detected for CaSMP17 (A and B), CaSMP1 (C and D), CaSMP20 (E and F), CaSMP9 (G and H), CaSMP3 (I and J), and CaSMP5 (K and L). Hoechst is shown in gray and each SMP in cyan. ft, foot; sf, shell field; sfe, shell field edge; tn, tentacles; ve, velar lobes; ls, larval statocyst; lk, larval kidney. Scale bars in each represent 100 μm.

**Figure S19**


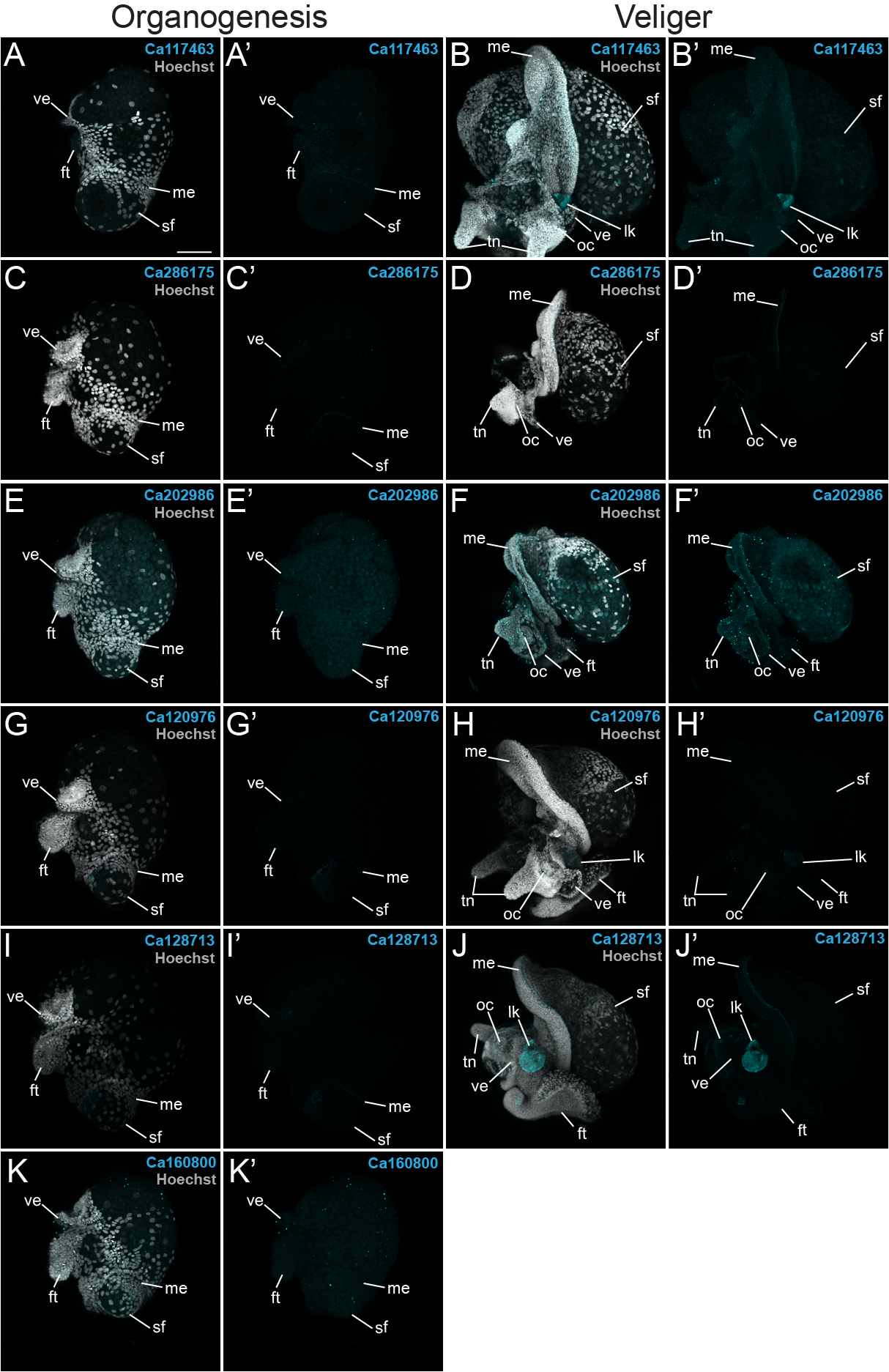


**Figure S19. SMPs without shell field expression in *C. atrasolea* at organogenesis and veliger stages.** Using *in-situ* hybridization chain reaction, mRNA expression was detected for Ca117463 (A and B), Ca286175 (C and D), Ca202986 (E and F), Ca120976 (G and H), Ca128713 (I and J), and Ca120800 (K). Hoechst is shown in gray and each SMP in cyan. ft, foot; sf, shell field; tn, tentacles; ve, velar lobes; lk, larval kidney. Scale bar represents 100 μm.

**Figure S20.**


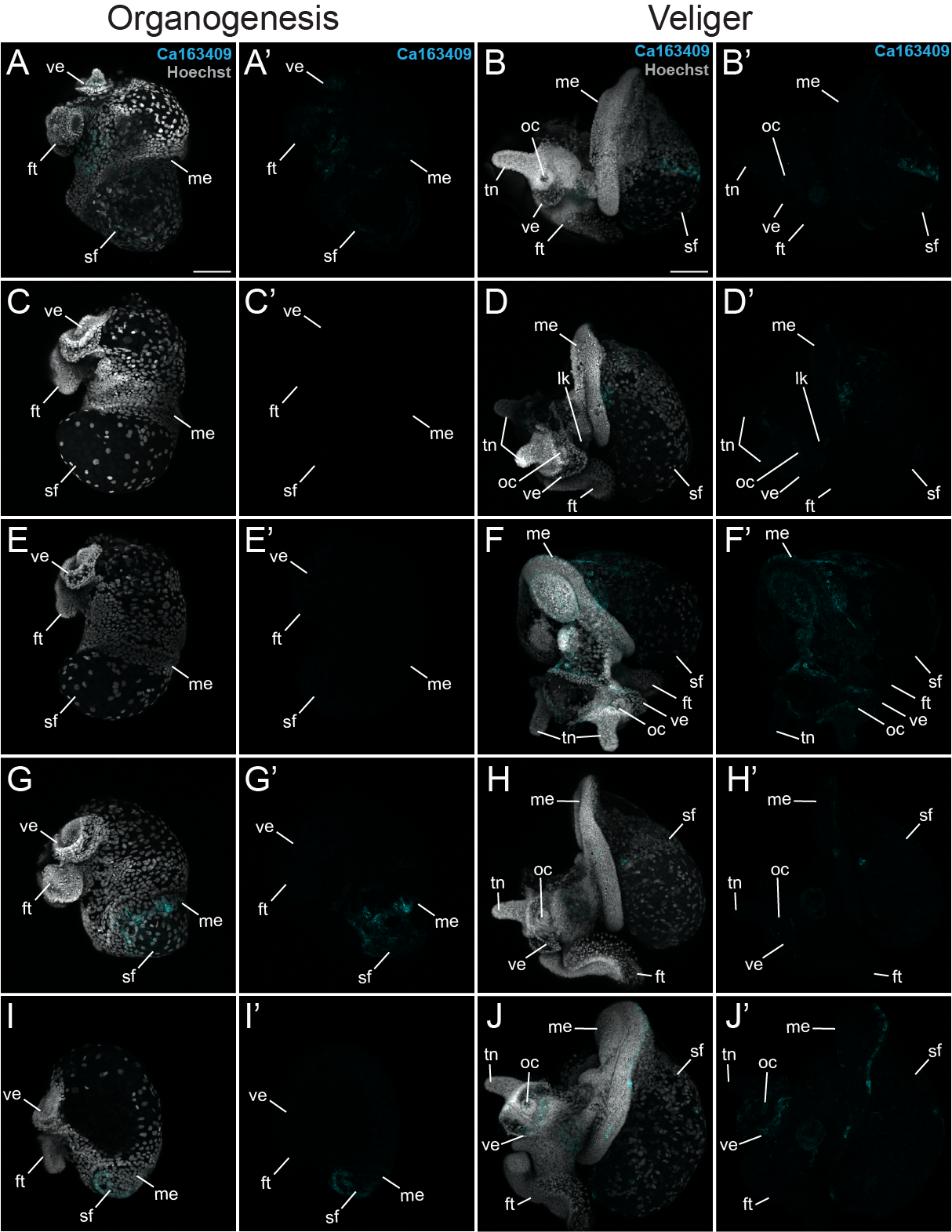


**Figure S20. Variation of *Ca163409* expression in *C. atrasolea* at organogenesis and veliger stages.** Using *in-situ* hybridization chain reaction, mRNA expression was detected for *Ca163409* with variation in patterns from faint shell field expression (A and B) to no expression (C and E) to stronger labeling of cells in the shell field (F, G, H, I, and J). Hoechst is shown in gray and each SMP in cyan. ft, foot; sf, shell field; tn, tentacles; ve, velar lobes; lk, larval kidney. Scale bar represents 100 μm.

**Figure S21.**


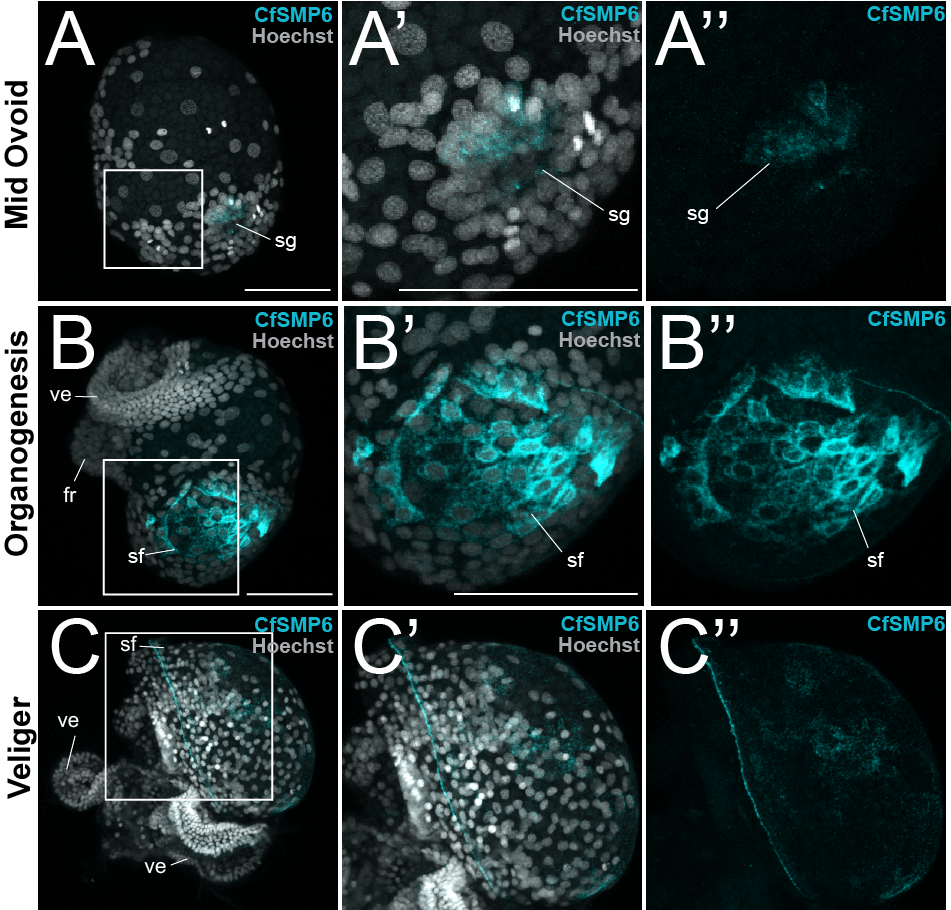


**Figure S21. Expression of *CfSMP6* expression during larval development in *C. fornicata*.** Using *in-situ* hybridization chain reaction, mRNA expression was detected for *CfSMP6* with during mid ovoid (A), organogenesis (B), and veliger (C). Hoechst is shown in gray and each SMP in cyan. ft, foot; sf, shell field; tn, tentacles; ve, velar lobes. Scale bar represents 100 μm.
